# Cohort profile: Walking for harm reduction through street engagement (WHiSE 2.0)

**DOI:** 10.1371/journal.pone.0354477

**Published:** 2026-07-24

**Authors:** Geena Verma, Muna Aden, Marie Hartman, Jennifer D. Walker, Kaela Pelland, Chris Mushquash, Savitri Persaud, Meghan Young, Holly Gauvin, Anita C. Benoit, Ontario Aboriginal HIV/AIDS Strategy, Elevate Northwestern Ontario

**Affiliations:** 1 Department of Health and Society, University of Toronto Scarborough, Scarborough, Ontario, Canada; 2 Ontario Aboriginal HIV/AIDS Strategy, Barrie, Ontario, Canada; 3 Department of Health Research Methods, Evidence, and Impact, McMaster University, Hamilton, Ontario, Canada; 4 Department of Psychology, Lakehead University, Thunder Bay, Ontario, Canada; 5 Elevate Northwestern Ontario, Thunder Bay, Ontario, Canada; 6 Dalla Lana School of Public Health, University of Toronto, Toronto, Ontario, Canada; 7 Women’s College Research and Innovation Institute, Women’s College Hospital, Toronto, Ontario, Canada; Virginia Commonwealth University School of Medicine, UNITED STATES OF AMERICA

## Abstract

Indigenous people using substances experience health disparities that are compounded by systemic inequities shaped by colonialism. The WHiSE 2.0 study aims to describe substance use patterns and harm reduction approaches among Indigenous people who use drugs in Thunder Bay, Sudbury, and Sault Ste Marie. A community-based research approach was used and guided by Indigenous leadership. Eligible study participants self-identified as Indigenous, lived in the study regions, and used substances within the last 3 months. Participants completed an interviewer-administered 1-hour questionnaire. Descriptive statistics were conducted to summarize participant demographic and cultural characteristics, impacts of colonization, substance use and health behaviours, and harm reduction knowledge, stratified by city. A total of 356 participants were enrolled: 173 in Thunder Bay, 101 in Sault Ste. Marie, and 82 in Sudbury. Most identified as First Nations with 93.1% in Thunder Bay, 76.8% in Sudbury, and 75.2% in Sault Ste. Marie. The percentage of participants across all sites with precarious sleeping situations was high, with 39.0% sleeping on the street, 28.3% staying in a shelter, and 23.3% couch surfing as well as 41.9% staying at a family or friend’s home. Across all sites, 43.3% of participants injected drugs, 31.5% ingested drugs, 92.1% smoked drugs, and 38.5% snorted or inhaled drugs. Importantly across all sites, 84.0% knew what harm reduction was, and 82.3% of respondents had ever had HIV screening test and 84.3% hepatitis C testing. Also, 71.3% of all participants engaged in ceremonies and 22.2% reported that cultural teachings affected their harm reduction practices. WHiSE 2.0 is the first prospective cohort study examining the harm reduction needs of Indigenous people using substances in northern Ontario. Through a culturally grounded, community-led study, urgent service gaps are highlighted and the importance of locally tailored harm reduction strategies rooted in Indigenous knowledge and leadership.

## Introduction

Walking for Harm Reduction through Street Engagement (WHiSE) 2.0 builds on the foundational 2020 WHiSE study, which explored harm reduction needs among street-affected Indigenous people who use drugs (PWUD) in Thunder Bay. Recognizing the need for broader geographic reach and deeper community engagement, in WHiSE 2.0 we expanded this work to include Sault Ste. Marie and Sudbury. The study aims to understand the local harm reduction needs, service use patterns, and priorities of Indigenous PWUD in three northern Ontario cities to inform and strengthen access to culturally safe services. Consistent with Indigenous research methodologies, which position relationality as a foundational principle [1–3], this study was in partnership with the Ontario Aboriginal HIV/AIDS Strategy (Oahas) and Elevate Northwestern Ontario (ENWO) and with academic researchers. These partnerships ensured that the research was grounded in strong, trust-based relationships with community-based organizations, which are critical to the project’s relevance and success.

The coordinating study site in the 2020 WHiSE study was ENWO in Thunder Bay where prior research reported a high proportion of people using opioids and a high number of PWUD [4,5]. Both groups are at a high risk for health issues and harms related to using substances [4,5]. The 2017 feasibility study for a supervised injection site (SIS) in Thunder Bay (n = 196), recruiting Indigenous participants (22%), found that 19% of people who inject drugs (PWID) shared needles and 36% shared other injection equipment [4]. Of the participants, 69% would use a SIS for access to hygienic and safe spaces, to prevent overdoses and to reduce the transmission of sexually transmitted and blood-borne infections (STBBI) [4]. However, Indigenous-specific data were not reported in the feasibility study. In the 2020 WHiSE study, 80.5% (n = 149) of Indigenous PWUD accessed a safe consumption site (SCS) for a safer space, drug use, and social and health services [6]. The data highlighted the urgent need to support evidence-based harm reduction and related services to meaningfully engage PWUD. This is a pressing issue given the closures of SCS on March 31, 2025, across Ontario [7,8].

Moreover, in the Thunder Bay District Health Unit (TBDHU), the rate of opioid overdose has increased over time and is higher than the provincial rate. Between 2005 and 2016, the crude rate of emergency department visits for opioid overdose increased from 32.9 to 53.4 per 100,000 people [9]. From 2016 to 2020, 78.9% of newly diagnosed HIV cases in the TBDHU were among Indigenous people, and the homeless or under-housed populations [10]. Many cases were linked to known individuals living with HIV, with transmission primarily occurring through sexual contact and needle sharing [10]. Consistent with this, the TBDHU declared a community HIV outbreak on June 25, 2019 [11], with transmission reported via sexual activity and sharing injection drug supplies. Comparable data for Sudbury and Sault Ste. Marie remain limited, highlighting a critical knowledge gap. Furthermore, the hepatitis C virus (HCV) burden remains largely understudied among Indigenous people [12–14]. In a recent study (1999–2018) in Ontario, 12.2% (n = 1,462) of status First Nations people had ever tested positive for HCV antibodies [15]. In a retrospective study (2010–2015) among 31 rural and remote First Nations communities in northwestern Ontario, the reported HCV incidence was 324.2 per 100,000 people [16]. Injection drug use (IDU) was the primary exposure category for both HIV and HCV among Indigenous populations [17,18]. Of note is that First Nations populations with HCV were characteristically different (e.g., higher proportion of females, younger age) from non-First Nations people with HCV highlighting the importance of understanding these differences to inform HCV preventative and therapeutic strategies that can adapt to women’s needs (e.g., reproductive outcomes of younger aged females, estrogen effects on the liver in post-menopausal women, family support as part of HCV care, concerns of child apprehension if accessing care) [14,19]. In general, females have been reported to be at a higher risk of HCV exposure through equipment and syringe sharing [20] most notably among Indigenous females [19]. These elevated risks are shaped by structural factors such as poverty, racism, marginalization, and the criminalization of substance use [12,17].

This cohort profile describes the WHiSE 2.0 study design, recruitment methods, and baseline participant characteristics. This study seeks to generate locally driven, evidence-based insights to inform harm reduction programming and other affiliated health and social services grounded in local Indigenous cultures which are led by and for Indigenous communities.

## Methods

### Study design and objectives

The WHiSE 2.0 observational cohort study uses an explanatory sequential mixed methods study design for data collection, but only the first round of data collection from the quantitative component will be reported. This project is guided by community-driven priorities, and the questionnaire was co-developed and underwent several months of review with the grant’s community and academic investigators. Pre-testing and piloting of the questionnaire also took place with Indigenous community members and research assistants aligned with survey design processes and with considerations to the total survey error (TSE) framework [21–23].

The proposed questionnaire was initially developed from operational definitions of key concepts important to this study such as harm reduction and street-affected and further refined for WHiSE 2.0. Operational terms include terms (i.e., domains) within definitions that can be measured (i.e., variables) or further defined (i.e., sub-domains) to then be measured. Pre-testing included a phase whereby the proposed questionnaire by ENWO and Oahas staff was discussed with potential study participants, and academic and community investigators. Feedback was provided on all existing domains with the possibility of adding or removing domains. Feedback was also obtained on the questions and answer options. These meetings occurred as group discussions. An additional pre-testing phase included administering the questionnaire to potential study participants who upon completing the questionnaire individually shared feedback on all aspects of the questionnaire (e.g., length, duration of completion, types of questions and answer options, comprehension) in small group discussions. The research assistants and those testing the questionnaire also evaluated ease of administering the questionnaire as well as skip patterns and branching for example when uploaded on REDCap. The piloting phase involved feasibility of administering the questionnaire to recruited study participants and continued evaluation of the questionnaire on REDCap by the research assistants (e.g., clarity and need for preamble and definitions in the questionnaire, feasibility of recruitment and screening process, sampling). The TSE framework was critical in guiding the elimination or minimization of observation and non-observation errors in the survey design process.

The descriptive objectives were to identify harm reduction practices, demand for and characteristics of harm reduction services, and rates of substance use and STBBI. The analytical objectives focus on examining associations between social, structural, and behavioural factors influencing substance use. Community engagement objectives include promoting harm reduction services and resources, conducting research training, building community relationships, and developing deliverables for diverse rights and interest-holders.

This paper focuses on the descriptive objectives investigated using the questionnaire to develop a cohort profile of Indigenous participants who use substances in Thunder Bay, Sudbury, and Sault Ste. Marie. There are three rounds of data collection with the questionnaire and our findings reflect the first round of data collection.

### Study setting and population

The study setting was northern Ontario with community investigator partners, ENWO and Oahas. ENWO is in Thunder Bay and Oahas satellite sites are in Thunder Bay, Sault Ste. Marie and Sudbury. Both community-based, not-for-profit organizations deliver onsite and outreach harm reduction and support services as well as programming and opportunities for people living with or affected by HIV. ENWO specifically delivers HIV and HCV health services along the cascade of care with an Elder providing support and programming to its clients [24]. Oahas delivers Indigenous grounded services, and HIV prevention and referral services with some satellite site-specific differences; for example, one site collaborates with a paramedicine program to deliver skin and soft tissue infection care while delivering their outreach program [25]. Each partner hosts WHiSE 2.0 research assistants ensuring a private space to administer the questionnaire and supporting administrative activities (e.g., managing honorarium, identifying Elders, and sharing study information with potential participants) to various degrees. According to PLOS One policy, the questionnaire on inclusivity in global research has also been completed (S2 File).

Eligible study participants included those who: (1) self-identified as First Nations, Métis, Inuk, or any combination of the three; (2) lived in Thunder Bay, Sudbury, or Sault Ste. Marie; (3) injected, inhaled/snorted, smoked, or ingested (oral consumption) substances within the past three months; (4) are aged 16 years or over; (5) understood English; and (6) provided informed consent. Individuals reporting using cannabis and alcohol only were excluded. Our study participants were also street-affected which we define as (a) individuals who do not have stable, permanent, or appropriate housing due to systemic or societal barriers and the individual’s financial, mental, cognitive, behavioural or physical challenges, and/or racism and discrimination; and (b) who may rely on the streets and an informal economy (e.g., under the table, working for cash, off the books) as well as emergency services (e.g., food banks, soup kitchens, drop-ins) to meet their basic needs [26,27].

Study participants received $33, refreshments, and a tobacco tie. In our study, a tobacco tie is prepared by adding tobacco leaves to the centre of a small square piece of cotton fabric. The cloth can vary in size, but ideally 4–6 inches permitted us to fold all ends of the cloth and tie the cloth with a strip of the fabric or a piece of yarn. Following local protocol, a tobacco tie was offered to each study participant typically with the left hand to ask for their assistance and guidance in the research study and to acknowledge that the knowledge they are sharing is sacred. Accepting the tobacco tie reflects an agreement to contribute to the best of their abilities. Participants in turn can use the tobacco tie when requesting support from Knowledge Carriers for example. Our community partners have relationships with diverse Knowledge Carriers who can provide support to study participants as clients when needed. Our study included a Knowledge Carrier as part of the research team who provided feedback on the study and regularly provided support outside of the study to our community partners’ clients who use substances. Also, a study participant may leave the study site and access cultural supports at a site not involved in this study using their tobacco tie.

### Sampling strategy and recruitment

Participants were recruited through purposive and snowball sampling in each city. Our sample size targets were 185 in Thunder Bay, 100 in Sudbury, and 100 in Sault Ste. Marie for each round of data collection. To obtain a confidence level of 95%, with a population proportion of 50%, a sample size of 385 is needed. The possibility of recruiting an additional 25 participants in Sudbury and Sault Ste. Marie, respectively, was also budgeted for. There is no information assessing the proportion of Indigenous people using substances, particularly in Sudbury and Sault Ste. Marie, so 50% was used to reach the minimum sample size for a questionnaire.

Recruitment strategies included distributing flyers and informal announcements during outreach shifts and group activities at ENWO and Oahas. Locally hired research assistants were partnered with local service organizations where harm reduction services are in geographically distinct regions in each of the cities. Research assistants hosted information sessions in each city to introduce the study and address questions. They informed potential participants of study procedures and scheduled times to screen for eligibility, obtain consent, and administer the questionnaire.

### Data collection

The questionnaire is administered and managed using REDCap (Research Electronic Data Capture) hosted at the University of Toronto [28,29]. REDCap is a secure, web-based software platform supporting data capture for research studies, providing 1) an intuitive interface for validated data capture; 2) audit trails for tracking data manipulation and export procedures; 3) automated export procedures for seamless data downloads to common statistical packages; and 4) procedures for data integration and interoperability with external sources. REDCap permitted offline and online data collection in community settings. Private spaces at each partner site were used to ensure confidentiality while administering the questionnaire.

The WHiSE 2.0 study began in November 2022, with data collection occurring in phases across sites with one research assistant per site. Questionnaires were administered in Thunder Bay from February 2023 to June 2024, in Sault Ste. Marie from May to November 2024, and in Sudbury from May to December 2024. Thunder Bay was the pre-testing and pilot site for the questionnaire where feedback was incorporated, and final adjustments were made to improve the questionnaire from format, layout, and to the structure and clarity of questions. Round 2 of data collection is in progress with 2 research assistants per study site.

### Questionnaire domains

The WHiSE 2.0 interviewer-administered questionnaire includes the following domains: demographics, cultural connection, drug use history, harm reduction knowledge and practices, access and attitudes toward supervised injection and consumption sites, HIV and HCV testing, and overdose prevention (S1 File). The questionnaire consists of 119 questions with sub-questions for some. While most questions are consistent across sites, some are tailored to reflect local contexts. All data were self-reported. Participants could select “Don’t know” or “Prefer not to answer” for questions, and open-ended responses are available when “Other” is selected. This manuscript reports on selected survey domains; further findings will be shared in future city-specific analyses.

### Demographic characteristics

Participants were categorized by key demographic characteristics, including Indigenous identity grouping and age. Sex at birth was reported as female, male or other. Gender identity was also collected along with type of partners. Living situations were assessed based on participants’ current sleeping arrangements, and sources of income were captured.

#### Cultural practices and impacts of colonization.

Cultural practices were assessed by asking participants whether they engaged in First Nations, Inuit, or Métis ceremonies. The impacts of colonization were explored through questions on intergenerational trauma such as experiences with residential schools, the Sixties Scoop, and the child welfare system. Additional impacts of colonization included discrimination in healthcare, workplaces, and the justice system.

#### Substance use and harm reduction.

Substance use was assessed by injecting, smoking, snorting or inhaling, and ingesting [oral consumption] practices within the past three months. Questions also included whether substances were used alone, with a friend, or with strangers, as well as in combination with alcohol or other substances. Data was also collected on overdose experiences. Overdose was being captured, but we acknowledge that the word “poisoning” may more accurately reflect participants’ experiences. Harm reduction knowledge as well as barriers were determined. Questions on methadone and naloxone use, and awareness of the Good Samaritan Drug Overdose Act were asked. The Act provides legal protection (i.e., arrest, charge, prosecution) for individuals who seek help while experiencing or observing an overdose or poisoning due to substance use. Protection includes leaving the scene, and charges of possessing controlled substances or breach of conditions regarding simple possession [30].

#### Health behaviours.

Participants were asked about their health behaviours such as HIV, HCV, and other STBBI testing, receiving antiretroviral therapy (ART), and their beliefs on the role of culture and increased support. Participants were also asked about having substance use-related injuries and whether they accessed services for these injuries.

### Ethics and data governance

Research ethics clearance was obtained from the University of Toronto Research Ethics Board (Protocol # 43512). All participants provided verbal and written informed consent. Participant confidentiality was strictly maintained by de-identifying all data and assigning unique identification (ID) codes. Consent forms and identifying information (i.e., honorarium login sheets, ID linking forms) are stored separately by the research assistants at the study sites. Research assistants mail consent forms and login sheets to the coordinating study site periodically if hard copies were used. Access to all documents is restricted to authorized personnel.

Aspects of OCAP® (Ownership, Control Access, Possession) [31] and the CARE (Collective benefit, Authority to control, Responsibility, and Ethics) principles [32] as well as select features from the Indigenous data framework of the Institute for Clinical Evaluative Sciences (IC/ES) [33] were used to establish the WHiSE 2.0 governance processes to promote community standards. For OCAP®, ownership (‘*O’*CAP) of the data was by the Indigenous community investigator, and control (O‘*C’*AP) of the data is that research questions and objectives investigated are community priorities. Access (OC*‘A’*P) of de-identified data is limited to the analyst on REDCap, and access and possession (OCA*‘P’*) by the nominated principal investigator on REDCap and the institutional SharePoint. The community investigators can access the data on SharePoint but prefer data summaries. For the CARE principles, in terms of collective benefit (‘*C’*ARE), our project was determined by Indigenous and allied leaders delivering harm reduction and training local community members as research assistants. Other anticipated benefits included promoting services by our community partners available to study participants as clients that are not contingent on participating in the research study. Also, for use of the data beyond our project objectives, an Indigenous advisory committee will be established to determine who uses the data and how the proposed work may inform programming and policies for harm reduction for example. Authority to control (C*‘A’*RE) follows control in OCAP. In terms of responsibility (CA‘*R’*E), our research team including staff is dedicated to improving the health outcomes of Indigenous people using substances and supporting action-oriented research. Importantly, our questionnaire has Indigenous-specific content and reflects the priorities of street-affected Indigenous people who use substances and was informed by the population for whom the research should benefit through the pre-testing and pilot testing of the questionnaire. For ethics (CAR‘*E’*), we are primarily an Indigenous research team who will assess the benefits, harms, and future uses of the data based on community values. From ICES, we will put into practice three processes, the first being that the research must be important to our community partners; Oahas and ENWO. Next the research must involve capacity building for Indigenous peoples, and we have successfully hired Indigenous persons as research assistants. Finally, the last process is to ensure that the community interprets the data and decides how the findings will be shared.

Development of the WHiSE data governance framework involved incorporating the principles. The research team met virtually monthly and over 2 days to discuss and review various data governance processes led by Indigenous interest- and rights-holders as well as by academic, clinical, and organizational entities [32–39]. The purpose of the framework is to manage and ensure the quality, security, and compliance of the study. It is a living document that establishes a set of processes and responsibilities to effectively manage data throughout the lifecycle of the research study. It will be used to guide data related decision-making for the study to meet the objectives with site-specific considerations. The framework covers the collection, storage, use, analysis, dissemination, retention and disposal of study data.

### Statistical analyses

Statistical analyses were performed using SAS version 9.4 (SAS Institute Inc.). Descriptive statistics were stratified by city to capture local variation. Categorical variables were summarized using frequencies and proportions. To protect confidentiality and reduce re-identification risk, cell sizes fewer than 5 were suppressed and marked as “ < 5,” with corresponding percentages removed. Ranges were presented where appropriate. Categories were not mutually exclusive; participants could select multiple responses when applicable. Missing data and answer options “Don’t know/ not sure /prefer not to answer” were minimal for each table and are indicated in below each respective table where applicable. Where a “other” answer option is available,“don’t know/ not sure/ prefer not to answer” were included. Participants were not removed from the analysis if they were unable to answer a few questions out of a total of 119 questions.

## Results

### Participant demographics

The WHiSE 2.0 questionnaire was completed by 356 study participants across three cities: Sault Ste. Marie (n = 101), Sudbury (n = 82), and Thunder Bay (n = 173) (Table 1). Overall, 84.3% identified as First Nations, specifically, 93.1% in Thunder Bay, 76.8% in Sudbury, and 75.2% in Sault Ste. Marie. 42.4% of participants were aged 40 year or over, 37.6% aged 30–39 years, and 19.1% were aged 18–29 years.

**Table 1:** Demographic characteristics of study participants stratified by city (n = 356).

	Sault Ste. Marie (n = 101), n (%)	Sudbury (n = 82), n (%)	Thunder Bay (n = 173), n (%)	Overall (n = 356), n (%)
Indigenous identity grouping				
First Nations	76 (75.2%)	63 (76.8%)	161 (93.1%)	300 (84.3%)
Métis	22 (21.8%)	16 (19.5%)	12 (6.9%)	50 (14.0%)
Inuk or Combination	* < 5	* < 5	0	6 (1.7%)
Age (years)				
18-29	15 (14.9%)	16 (19.5%)	37 (21.4%)	68 (19.1%)
30-39	37 (36.6%)	34 (41.5%)	63 (36.4%)	134 (37.6%)
40+	48 (47.5%)	32 (39.0%)	71 (41.0%)	151 (42.4%)
Sex at birth				
Female	51 (50.5%)	35 (42.7%)	91 (52.6%)	177 (49.7%)
Male	50 (49.5%)	47 (57.3%)	80 (46.2%)	177 (49.7%)
Gender identity				
Woman	49 (48.5%)	33 (39.3%)	91 (52.6%)	173 (48.6%)
Man	49 (48.5%)	46 (54.8%)	79 (45.7%)	169 (47.5%)
2-Spirit, Trans woman Non-binary	* < 5	4-8	* < 5	9 (2.5%)
Type of Partner**				
Regular	32 (31.7%)	26 (31.7%)	63 (36.4%)	121 (34.0%)
Casual	5 (5.0%)	5 (6.1%)	11 (6.3%)	21 (5.9%)
Other	* < 5	0	* < 5	* < 5
Current sleeping situation**				
House/apartment that I rent	17 (16.8%)	12 (14.6%)	41 (23.7%)	70 (19.7%)
Family/friend’s house/ apartment	13 (12.9%)	30 (36.6%)	106 (61.3%)	149 (41.9%)
Cluster unit/tent city	0 (0.0%)	15 (18.3%)	24 (13.9%)	39 (11.0%)
Couch surfing	14 (13.9%)	10 (12.2%)	59 (34.1%)	83 (23.3%)
Shelter	22 (21.8%)	16 (19.5%)	63 (36.4%)	101 (28.3%)
On the street	53 (52.5%)	44 (53.7%)	42 (24.3%)	139 (39.0%)
Other^a^	6 (5.9%)	11 (13.4%)	21 (12.1%)	38 (10.7%)
Sources of income**				
Full/part-time work	* < 5	14-18	14-18	32 (9.0%)
Government^b^	92 (91.1%)	77 (93.9%)	147 (85.0%)	316 (88.8%)
Drug transactions^c^	31 (30.7%)	63 (76.8%)	135 (78.0%)	229 (64.3%)
Family/partner	* < 5	14-18	120-124	138 (38.8%)
Panhandling	25 (24.8%)	33 (40.2%)	53 (30.6%)	111 (31.2%)
Sex work	19 (18.8%)	7 (8.5%)	17 (9.8%)	43 (12.1%)
Selling items	24 (23.8%)	31 (37.8%)	97 (56.1%)	152 (42.7%)
Shoplifting	40 (39.6%)	21 (25.6%)	53 (30.6%)	114 (32.0%)
Under the table work	15 (14.9%)	17 (20.7%)	70 (40.5%)	102 (28.7%)
Honorariums/E-transfer	* < 5	14-18	104-108	127 (35.7%)
Other^d^	72 (71.3%)	42 (51.2%)	20 (11.6%)	134 (37.6%)

Non-binary=neither man nor woman, Trans woman = male to female

a Other current sleeping situations include owning a home, renting a hotel room, residing in a single-room occupancy unit, owning an apartment, or other. Responses of “Don’t know” or “Prefer not to answer” were also included.

b Government sources of income include Employment Insurance, Ontario Works, Ontario Disability Support Program, Pension or Provincial Health and Disability Income.

c Drug-related transactions include income from middling to selling drugs.

d Other sources of income include loansharking, boosting, other sources, or having no income at the time of the study. Responses of “Don’t know” or “Prefer not to answer” were also included.

* Values below 5 have been suppressed and replaced with <5 to ensure confidentiality. Percentages for suppressed values have also been removed for privacy reasons.

**Groups within some categories are not mutually exclusive thus respondents can indicate all responses that apply.

Missing data: Sault Ste Marie (n = 1 question) and Thunder Bay (n = 1–2 questions) for some categories.

Participants reported a range of current sleeping situations (Table 1). The most common locations included sleeping at a friends’ or family member’s house or apartment (41.9%) and sleeping on the street (39.0%). Other temporary arrangements included staying in shelters (28.3%), couch surfing (23.3%), and house or apartment that I rent (19.7%).

Participants reported diverse income sources (Table 1). Only 9.0% earned income through full- or part-time work, while most (88.8%) relied on government sources. Other sources of income included drug transactions (64.3%), selling items (42.7%), family or partner support (38.8%), honorarium or e-transfers (35.7%), shoplifting (32.0%), panhandling (31.2%), and under-the-table work (28.7%). Approximately 37.6% reported engaging in loansharking, boosting, other informal sources, or having no income.

### Cultural characteristics and impacts of colonization

A total of 71.3% of participants engaged in First Nations, Inuit, or Métis ceremonies (Table 2). Most participants (73.3%) knew someone who had gone missing or was murdered and 76.4% identified as intergenerational residential school survivors. Many participants reported involvement with the child welfare system (CWS), either as a child (57.6%) or a parent (40.2%). Discrimination was reported in the healthcare system (64.6%), justice system (64.0%), the workplace (39.9%), and the education system (62.9%).

**Table 2:** Cultural characteristics and impacts of colonization of study participants stratified by city (n = 356).

	Sault Ste. Marie (n = 101), n (%)	Sudbury (n = 82), n (%)	Thunder Bay (n = 173), n (%)	Overall (n = 356), n (%)
Practice First Nations, Inuit or Métis ceremonies	76 (75.2%)	58 (70.7%)	120 (69.4%)	254 (71.3%)
Intergenerational school survivor	72 (71.3%)	51 (62.2%)	149 (86.1%)	272 (76.4%)
Someone I know went missing or was murdered	73 (72.3%)	50 (61.0%)	138 (79.8%)	261 (73.3%)
Historical trauma^a^	17 (16.8%)	8 (9.8%)	12 (6.9%)	37 (10.4%)
CWS as a parent	53 (52.5%)	32 (39.0%)	58 (33.5%)	143 (40.2%)
CWS as a child	62 (61.4%)	37 (45.1%)	106 (61.3%)	205 (57.6%)
Healthcare discrimination	63 (62.4%)	52 (63.4%)	115 (66.5%)	230 (64.6%)
Workplace discrimination	54 (53.5%)	38 (46.3%)	50 (28.9%)	142 (39.9%)
Justice discrimination	74 (73.3%)	54 (65.9%)	100 (57.8%)	228 (64.0%)
Education discrimination	73 (72.3%)	47 (57.3%)	104 (60.1%)	224 (62.9%)
Other	21 (20.8%)	15 (18.3%)	5 (2.9%)	41 (11.5%)

Child welfare system = CWS

^a^Historical trauma includes being a residential school survivor, a parent during the sixties scoop, and/or a child taken during the sixties scoop.

### Substance use behaviors

Substance use practices in the past three months have varied by city (Table 3). Drug injections were reported by 43.3% of the overall participants with 70.7% in Sudbury, 40.5% in Thunder Bay, and 25.7% in Sault Ste. Marie. Ingesting drugs was reported by 31.5% overall, with the highest rates in Sudbury (58.5%), followed by Thunder Bay (34.1%), and Sault Ste. Marie (5.0%). Smoking drugs was high across all sites: 96.0% in Sault Ste. Marie, 91.3% in Thunder Bay, and 89.0% in Sudbury. Snorting or inhaling substances was reported by 38.5% overall: 52.4% in Sudbury, 48.0% in Thunder Bay, and 10.9% in Sault Ste. Marie.

**Table 3:** Substance use behaviours of study participants stratified by city (n = 356).

	Sault Ste. Marie (n = 101), n (%)	Sudbury (n = 82), n (%)	Thunder Bay (n = 173), n (%)	Overall (n = 356), n (%)
Inject drugs				
In the last 3 months	26 (25.7%)	58 (70.7%)	70 (40.5%)	154 (43.3%)
And drink alcohol	6 (23.1%)	28 (48.3%)	54 (77.1%)	88 (57.1%)
And drink other products	0 (0.0%)	7 (12.1%)	8 (11.4%)	15 (9.7%)
Alone	23 (88.5%)	52 (89.7%)	60 (85.7%)	135 (87.7%)
With buddy system	18 (69.2%)	43 (74.1%)	55 (78.6%)	116 (75.3%)
With strangers	13 (50.0%)	35 (60.3%)	36 (51.4%)	84 (54.5%)
Ingest drugs	5 (5.0%)	48 (58.5%)	59 (34.1%)	112 (31.5%)
In the last 3 months				
And drink alcohol	* < 5	20-24	46 (78.0%)	71 (63.4%)
And drink other products	0 (0.0%)	10 (20.8%)	12 (20.3%)	22 (19.6%)
Alone	* < 5	30-34	45 (76.3%)	82 (73.2%)
Smoke drugs	97 (96.0%)	73 (89.0%)	158 (91.3%)	328 (92.1%)
In the last 3 months				
And drink alcohol	26 (26.8%)	35 (47.9%)	131 (82.9%)	192 (58.5%)
And drink other products	4 (4.1%)	14 (19.2%)	16 (10.1%)	34 (10.4%)
Alone	75 (77.3%)	68 (93.2%)	146 (92.4%)	289 (88.1%)
Snort or inhale drugs	11 (10.9%)	43 (52.4%)	83 (48.0%)	137 (38.5%)
In the last 3 months				
And drink alcohol	6 (54.5%)	21 (46.7%)	63 (75.9%)	90 (65.7%)
And drink other products	0 (0.0%)	14 (32.6%)	20 (24.1%)	34 (24.8%)
Alone	10 (90.9%)	38 (88.4%)	64 (77.1%)	112 (81.8%)
Use more than one drug at a time	64 (63.4%)	71 (86.6%)	136 (78.6%)	271 (76.1%)
Ever overdose	84 (83.2%)	60 (73.2%)	98 (56.6%)	242 (68.0%)
Most recent overdose				
Last week or month	9 (10.7%)	6 (10.0%)	26 (26.5%)	41 (16.9%)
Last 3 months	10 (11.9%)	11 (18.3%)	13 (13.3%)	34 (14.0%)
Last 4–5 months	9 (10.7%)	* < 5	5-9	16 (6.6%)
Last 6–12 months	19 (22.6%)	14 (23.3%)	7 (7.1%)	40 (16.5%)
1 year ago or more	37 (44.0%)	26 (43.3%)	45 (45.9%)	108 (44.6%)

* Values below 5 have been suppressed and replaced with <5 to ensure confidentiality. Percentages for suppressed values have also been removed for privacy reasons.

Missing data including prefer not to answer and don’t know: Sault Ste Marie (n = 1–2 questions), Sudbury (n = 2 questions), and Thunder Bay (n = 2–6 questions) for some categories.

Ever having reported an overdose varied among study participants from 83.2% in Sault Ste. Marie, 73.2% in Sudbury, to 56.6% in Thunder Bay with 68.0% across all sites. Use of the buddy system when injecting was highest in Thunder Bay, 78.6%, followed by 74.1% in Sudbury, and 69.2% in Sault Ste. Marie. Injecting alone was reported by 89.7% in Sudbury, 88.5% in Sault Ste. Marie, and 85.7% in Thunder Bay. Concurrent use of multiple substances was high: 86.6% in Sudbury, 78.6% in Thunder Bay, and 63.4% in Sault Ste. Marie. Having experienced a lifetime overdose with regional variations was self-reported by 68.0% of study participants.

### Harm reduction

Harm reduction use and knowledge was assessed across each city (Table 4). Across all sites, 84.0% of participants reported knowing what harm reduction is, and 94.8% knew where to access harm reduction services. Barriers to practicing harm reduction were reported by 29.3% of participants in Sudbury, 25.4% in Thunder Bay, and 10.9% in Sault Ste. Marie. Barriers to harm reduction included access to harm reduction such as a lack of transportation, being unaware or barred from locations; requiring childcare; privacy and confidentiality of locations; having a conflict, feeling unsafe or disliking harm reduction staff; and cultural or other belief systems not aligned with harm reduction principles. Indigenous teachings influenced harm reduction practices for 28.0% of participants in Sudbury, 27.7% in Sault Ste. Marie, and 22.5% in Thunder Bay. Overall, 96.1% of participants had heard of methadone. Methadone use was highest in Sudbury at 51.2% compared to 28.9% in Thunder Bay and 27.7% in Sault Ste. Marie. Naloxone use also varied with 82.2% in Sault Ste. Marie, 78.0% in Sudbury, and 71.2% in Thunder Bay. Awareness of the Good Samaritan Drug Overdose Act was 88.1% in Sault Ste. Marie, 69.4% in Thunder Bay and 67.1% in Sudbury.

**Table 4:** Harm reduction understandings of study participants stratified by city (n = 356).

	Sault Ste. Marie (n = 101), n (%)	Sudbury (n = 82), n (%)	Thunder Bay (n = 173), n (%)	Overall (n = 356), n (%)
Know what harm reduction is	83 (82.2%)	74 (90.2%)	142 (82.1%)	299 (84.0%)
Know where to go for harm reduction	100 (99.0%)	76 (92.7%)	161 (93.1%)	337 (94.7%)
Barriers to practicing harm reduction	11 (10.9%)	24 (29.3%)	44 (25.4%)	79 (22.2%)
Teachings affect harm reduction practices	28 (27.7%)	23 (28.0%)	39 (22.5%)	90 (25.3%)
Ever heard of methadone	99 (98.0%)	78 (95.1%)	165 (95.4%)	342 (96.1%)
Ever used methadone	28 (27.7%)	42 (51.2%)	50 (28.9%)	120 (33.7%)
Ever used Naloxone	83 (82.2%)	64 (78.0%)	124 (71.2%)	271 (76.1%)
Aware of Good Samaritan Drug Overdose Act	89 (88.1%)	55 (67.1%)	120 (69.4%)	264 (75.1%)

* Values below 5 have been suppressed and replaced with <5 to ensure confidentiality. Percentages for suppressed values have also been removed for privacy reasons.

Missing data: Sault Ste Marie (n = 1–2 questions), Sudbury (n = 3–10 questions), and Thunder Bay (n = 2–7 questions) for some categories.

### Health and social service use

Across all sites, 82.3% of participants had ever been tested for HIV, 84.3% for HCV, and 72.5% for other STBBI (Table 5). HIV diagnoses were reported by 9.6% of participants, 44.0% were diagnosed with HCV, and 17.8% with another STBBI. A higher proportion of participants in Sudbury (54.5%) and Thunder Bay (45.0%) reported an HCV diagnosis compared to Sault Ste. Marie (34.1%). Testing for other STBBI also differed across sites with 81.2% tested in Sault Ste. Marie, 75.1% in Thunder Bay, and 56.1% in Sudbury. Belief that more cultural activities in the neighbourhood would support drug use specific support varied across cities. A high proportion of participants also reported substance use–related injuries or illness (62.1% overall). The use of services for these injuries or illnesses also differed across sites with 60.0% in Sudbury, 58.4% in Sault Ste. Marie, and 35.8% in Thunder Bay.

**Table 5:** Health and service use behaviours of study participants stratified by city (n = 356).

	Sault Ste. Marie (n = 101), n (%)	Sudbury (n = 82), n (%)	Thunder Bay (n = 173), n (%)	Overall (n = 356), n (%)
Ever been tested …				
HIV	81 (80.2%)	64 (78.0%)	148 (85.5%)	289 (82.3%)
HCV	85 (84.1%)	66 (80.5%)	149 (86.1%)	300 (84.3%)
Other STBBI	82 (81.2%)	46 (56.1%)	130 (75.1%)	258 (72.5%)
Ever been diagnosed … (among those tested)				
HIV	* < 5	4-8	16-20	28 (9.6%)
HCV	29 (34.1%)	36 (54.5%)	67 (45.0%)	132 (44.0%)
Other STBBI	9 (11.0%)	11 (23.9%)	26 (20%)	46 (17.8%)
Received ART (among those with an HIV diagnosis)	* < 5	4-8	16-20	24 (85.7%)
Believe more cultural neighborhood activities leads to …				
More general support	96 (95.0%)	63 (76.8%)	144 (83.2%)	299 (85.1%)
Substance use support	88 (87.1%)	49 (59.8%)	117 (67.6%)	254 (71.3%)
Ever had substance use related injuries or illness	84 (83.2%)	57 (69.5%)	80 (46.2%)	221 (62.1%)
Ever used services for substance use related injury or illness	59 (58.4%)	50 (60.0%)	62 (35.8%)	171 (48.0%)

Antiretroviral therapy = ART, Hepatitis C virus = HCV, Sexually transmitted blood borne infections = STBBI

* Values below 5 have been suppressed and replaced with <5 to ensure confidentiality. Percentages for suppressed values have also been removed for privacy reasons.

Missing data including prefer not to answer and don’t know: Sault Ste Marie (n = 1–7 questions), Sudbury (n = 2–10 questions), and Thunder Bay (n = 2–21 questions) for some categories.

## Discussion

### Summary of findings

Much of the existing research on harm reduction has focused on large urban centers in British Columbia [40–42] and Ontario [43–45]. A recent ethnographic study in Sudbury highlighted SCS as a critical part of harm reduction interventions and since then their closures have increased exposures to the harms and risk experienced by PWUD [46]. WHiSE 2.0 is the first quantitative prospective study examining harm reduction needs and experiences in smaller, historically underserved cities like Thunder Bay, Sudbury, and Sault Ste. Marie in Northern Ontario.

Our study participants (n = 356) reported a range of precarious sleeping situations highlighting hidden homelessness (i.e., 41.9% friend’s and family’s home, 23.3% couch surfing) and temporary sleeping situations (i.e., 39.0% on the street, 28.3% in shelters). Only a fifth of study participants (19.7%) were able to rent a house or apartment. For income, 88.8% of participants relied on government sources (e.g., employment insurance, Ontario Works, and Ontario Disability Support Program) followed by drug transactions (64.3%), selling items (42.7%) and support from their family or a partner (38.8%). Colonial determinants of health such as being an intergenerational school survivor (76.4%), knowing someone that went missing or was murdered (73.3%), being involved with the child welfare system as a parent or child as well as a range of discrimination experiences (~40–50%) were reported. These determinants may have impacted their socio-economic realities and health behaviours including substance use.

Substance use behaviours included injecting (43.3%), oral ingestion/consumption (31.5%), and snorting (38.5%), but smoking (92.1%) was consistently reported to be highest in all three cities. Importantly, for all three cities, 94.7% of study participants knew where to go for harm reduction and 82.3% underwent screening test for HIV, 86.1% were screened for HCV, and 72.5% for other STBBI. In addition, 71.3% of participants practiced First Nations, Inuit or Métis ceremonies and reported that more cultural neighbourhood activities would further contribute to greater substance use-specific supports.

### Regional differences in substance use and health behaviours

Substance use practices and experiences differed by city. Smoking drugs was high across all three cities, particularly in Sault Ste. Marie for our participants, where it took the top spot for opioid-related mortality several times between 2020–2024 [47,48]. Among all three cities, participants in Sudbury reported the highest proportions of injecting, and snorting (i.e., inhaling) drugs. These regional variations align with provincial surveillance: Ontario Drug Policy Research Network’s Ontario Opioid Indicator Tool shows that Northern public health units (Algoma, Sudbury & Districts, and Thunder Bay District) consistently experience among the highest opioid-toxicity harms in Ontario [49].

Slightly lower rates of STBBI (i.e., HIV, HCV, other) screening were reported by participants in Sudbury compared to the other cities. However, overall STBBI screening was high for HIV and HCV. Thus, we noted an overall rate of 9.6% (n = 28 out of 289 participants) of Indigenous people were diagnosed with HIV across our 3 sites with a range of 5.5–6.9% (n = 16–20 participants) in Thunder Bay. We did not capture diagnosis dates in our data collection so direct comparisons with TBDHU are not possible. Nevertheless, despite our relatively smaller sample size, we have an important representation of Indigenous people living with HIV in our study to inform future decision-making. Our results are consistent with earlier reporting by the TBDHU from 2016 to 2020 showing that 78.9% of HIV diagnosis were among Indigenous people and individuals with housing security [10]. A closer review of those case numbers shows that this percentage reflected 48 out of 60 cases out of approximately 158,000 individuals aged 15–59 years ^10^.

These regional variations have implications for tailored service planning. For example, Sudbury may benefit from enhanced access to a broader range of harm reduction supplies for substance use and increased access to culturally safe STBBI testing and treatment. While Sault Ste. Marie may require expanded naloxone distribution, access to safe consumption sites, and drug testing. These distinctions reinforce the need for localized harm reduction models reflecting specific geographic and cultural contexts [50,51].

### Intersecting harm reduction with healthcare

Close to half of our study participants (42.4%) were aged 40 years and over. Age-specific considerations may be supported by having increased collaboration between harm reduction initiatives and other health and social services to address the complex and evolving needs of people who use substances [51–53]. We could have more focused questions on the intersection of aging adults (65 + years) and harm reduction needs in future iterations of the WHiSE study. Questions on age-related health screenings such as routine cancer screening (e.g., breast cancer at age 40 years, colorectal cancer at age 50 years), cardiovascular health conditions, and geriatric-specific health needs could be considered given the lower health service access rates among PWUD [54–62]. More specifically, the incidence of cardiovascular disease has been associated with intravenous drug use as well as the use of methadone [60] and among our participants, 33.7% of participants reported ever using methadone and 43.3% injected drugs in the last 3 months.

Further evidence of the importance of enhanced knowledge and training in the application of harm reduction principles within healthcare systems could be beneficial since healthcare discrimination has been reported by 64.6% of our study participants. Previous research highlighted that 40% of PWUD avoided accessing healthcare services [63,64]. Indigenous PWUD in Vancouver’s inner city described racist and discriminatory healthcare and service provider encounters that prompted delayed and avoidance of healthcare services [65]. Additional research conducted with PWUD highlighted that the lack of negative experiences was considered a positive experience in the healthcare setting [66] meaning that baseline expectations were met versus specific positive experiences that could strengthen provider-client interactions [67].

### Culture in harm reduction

Most participants (84.0%) understood harm reduction and knew where to access these services (94.7%). Among the barriers against practicing harm reduction, cultural teachings were reported by 25.3% of participants. Awareness alone on harm reduction is insufficient without community-driven, trauma-informed and culturally safe delivery of harm reduction which has been used to address discrimination in the healthcare setting [53,68]. Specifically, we know that cultural interventions have been shown to improve wellness as well as reduce or eliminate substance use including reducing experiences of stigma [69] which can be nested within healthcare settings from community health centres and hospitals. This is important given our participants accessed cultural services (71.3%) and stated that more cultural activities in their neighbourhoods would create more opportunities for general support (85.1%) and targeted support towards substance use (71.3%). Our community partners, ENWO and Oahas, promote low-barrier participation in cultural activities and ceremony along with access to onsite and outreach harm reduction [24,25]. However they are experiencing increasing demands on their resources given that as a public health approach harm reduction is under attack and facing government restrictions [24,25].

Indigenizing harm reduction may also mitigate the stigma of practicing culture alongside harm reduction [52,70–72] by recognizing the compounding effects of colonial harms and its role in harmful substance use [52,70,73]. Colonial harms have been reported (e.g., intergenerational school survivor, systemic discrimination [i.e., healthcare, workplace, justice, and education], child welfare system) by our study participants and more work is needed to understand the association between cultural and harm reduction practices in contributing to redressing colonial harms.

Our research identifies critical areas for future inquiry, from the age-related concerns of PWUD, the long-term impacts of culturally grounded harm reduction and community-designed interventions in ways that reflect local needs and strengths.

### Socio-economic realities shaped by colonial harms

Socio-economic insecurities stem from the root determinants of health: colonialism, colonial ideologies (e.g., white supremacy, christianity, patriarchy, individualism) and colonial governance (e.g., reserve, settlements, capitalism) shaping Indigenous peoples’ lived experiences [74,75]. These insecurities were widespread among our participants, with many relying on informal income sources and lacking access to stable housing. These findings also echo prior literature that highlights housing and income insecurity as foundational determinants of health for Indigenous PWUD [69,73,76].

Pathways to homelessness or houselessness often involves poverty, mental illness, substance use, and housing shortages, these challenges are further compounded for Indigenous peoples by the lasting impacts of colonialism, including systemic racism, cultural oppression, and the dispossession of land [40]. Future acts or laws that broaden eviction powers or penalize landlords who do not evict tenants suspected of drug activity may increase housing instability by prompting rapid or pre-emptive evictions and discouraging harm reduction [77]. These dynamics underscore the need for culturally safe rights-based housing which prioritizes harm reduction and treatment readiness over displacement [69,73,76].

### Strengths and limitations

WHiSE 2.0 successfully enrolled 356 Indigenous study participants who were using substances across Thunder Bay, Sudbury, and Sault Ste. Marie. The study’s northern geographic coverage intends on supporting localized harm reduction programming and related social and health services. Its community-based approach fosters trust, reflects real-world conditions, and enhances the relevance of findings for Indigenous-led service delivery. This is also the first prospective study that will span the closure of the SCS across Ontario with the 2020 WHiSE study and the first round of WHiSE 2.0 data collected prior to their closures and a second and third round of data collection ending between May to August 2026 and early 2027. This study also includes a mixed methods analysis and although qualitative data is not reported here, future reporting of findings will expand on the quantitative findings based on priorities identified by study participants in the qualitative discussions.

Limitations include the use of self-reported data, which may be subject to recall or social desirability bias. However, this limitation was mitigated by building strong rapport with community partners and creating a culturally safe, confidential research environment to encourage responses that are as accurate as can be recollected. Also, local research assistants who were Indigenous or had experience working with Indigenous communities were hired. The data are currently cross-sectional and descriptive, limiting causal interpretation. Future waves of data collection will allow for inferential cross-sectional analyses to understand changes over time. Additionally, recruitment through community organizations may introduce selection bias and limit generalizability to individuals not engaged with services. We recruited a high percentage of participants aged 40 years and over which may reflect the fact that many organizations do not offer services to those aged 16 years and under. Also, they may not offer a wide array of services that are youth-specific (e.g., 16–24 years) which is typical for harm reduction programming to be more adult-centred given its origins [78–80]. Moreover, the distribution of study participants based on their methods for consuming substances may also have been impacted by the recruitment spaces.

### Conclusion

WHiSE 2.0 has contributed to our understanding of the harm reduction needs of Indigenous PWUD in Northern Ontario as well as their determinants of health. By reaching, enrolling, and retaining a large cohort of participants, the study has laid a strong foundation for future research and intervention development to improve health outcomes. As this is the first WHiSE 2.0 cohort paper, ongoing data collection across the three cities will deepen our understanding of the evolving harm reduction needs and experiences of Indigenous PWUD and inform harm reduction efforts grounded in Indigenous self-determination. Our expanded recruitment strategies will also contribute to this while continuing to include organizations that deliver harm reduction services, those focused on recovery and treatment only, and through outreach services. Moreover, the community-based research approach has ensured that the efforts are deeply rooted in the local contexts of the sites involved, thereby increasing the potential impacts of study outcomes. Moving forward, the second and third round of data collection will provide an opportunity to build on our findings, enabling more detailed analyses and fostering an environment where evidence-based policies can thrive, leading to substantial impacts in public health, harm reduction, across our study sites in Northern Ontario.

## Supporting information

S1 File WHiSE 2.0 survey. (PDF)

S2 File Inclusivity in global research questionnaire. (DOCX)

## Abbreviations:

ART: Antiretroviral therapy
CWS: Child welfare system
ENWO: Elevate Northwestern Ontario
HCV: Hepatitis C Virus
HIV: Human Immunodeficiency Virus
IDU: Injection drug use
Oahas: Ontario Aboriginal HIV/AIDS Strategy
OHTN: The Ontario HIV Treatment Network
PWID: People who inject drugs
PWUD: People who use drugs
REDCap: Research Electronic Data Capture
SAS: Statistical Analysis System
SCS: Safe consumption site
SIS: Supervised injection site
STBBI: Sexually transmitted blood-borne infections
TBDHU: Thunder Bay District Health Unit
WHiSE: Walking for Harm Reduction through Street Engagement
