# Supplementary material for: Cohort profile: Walking for harm reduction through street engagement (WHiSE 2.0)
Source: PLoS One. 2026 Jul 24;21(7):e0354477. doi: 10.1371/journal.pone.0354477 (PMC13399352; doi:10.1371/journal.pone.0354477)
Supplement: S1 File — (PDF) [file pone.0354477.s001.pdf]

WHiSE 2.0 Survey

Please complete the survey below.

Thank you!

WHiSE 2.0 Participant Informed Consent Form (Survey)

Project Title: Walking for Harm Reduction through Street Engagement (WHiSE 2.0)

Co-Principal Investigator:
Anita C. Benoit, Assistant Professor, Department of Health and Society, University of Toronto Scarborough, Toronto, Ontario- anita.benoit@utoronto.ca (647) 601-4566

Co-Principal Investigator:
Holly Gauvin, Executive Director, Elevate NWO, Thunder Bay, Ontario- hgauvin@elevatenwo.org, (807) 345-1516 ext.224

Co-Principal Investigator:
Meghan Young, Executive Director, Oahas, Toronto, Ontario-Meghan@oahas.org, (647) 210-0430

Funder: Canadian Institutes of Health Research

Background

What is this research study about?
This research study wants to know what street-affected Indigenous people who use drugs want in harm reduction services and whether there are Indigenous-specific needs. We also want to know what kind of harm reduction services (for example from safer drug use to testing for infections) are needed which partly involves learning more about what kind of drugs are being used because some services are drug-specific.

Why are you being asked to participate?
You are being asked to participate in this research study because you self-identified as being street-affected and an Indigenous person who has injected, smoked, inhaled/snorted, or orally taken prescribed or non-prescribed drugs in the last three months, currently living in Thunder Bay, Sudbury or Sault Ste Marie, Ontario, you are 16 years of age or older, and you speak and understand English.

What are you being asked to do?
You are being asked to go over this consent form (15 minutes) and complete a 40-minute survey with an interviewer on a tablet that has questions on your age, Indigenous grouping, language, sleeping situation, employment, and your culture. Other questions that will be asked are on your drug use, whether you get tested for infections, and on your knowledge and access of harm reduction and supervised injection sites. If you don't want to complete the survey after going over this consent form that's fine.

It is important that you know that you don't have to be part of this study to have access to harm reduction services such as new equipment for drug use, training on how to use naloxone kits, and any other information that you might want for yourself. Your access to any services at any sites where this study is taking place will not be impacted in any way whether you choose to participate or not. While completing the survey, we will offer you refreshments and you can still have access to refreshments if you don't want to complete the survey.

Will you be compensated for you time?

If you choose to participate in this study, to thank you for your time and for sharing your knowledge with us, we will provide you with an honorarium of $25.

What are the benefits of participating?
You may or may not benefit from participating in this research study. The study findings may inform policies and procedures for harm reduction services delivered in your city that might contribute to creating a culturally safer space for Indigenous people who substances.

What are the risks of participating?
If you choose to participate in this study, the cost of time may be an inconvenience to you. There are no known risks to your physical health from participating in this study (i.e., completing a survey). There may be risks to your emotional health when talking about and reflecting on your own experiences of drug use because this is a very personal issue. There might be other questions that sadden you or make you uncomfortable. Elders and trained Indigenous and non-Indigenous counselors, if you prefer, will be available to help you if you need to speak to someone. They are not involved in this study. Your participation is voluntary and you can decide to not answer any questions that upset you, or to end your participation at any time. There may be other unforeseen risks to participating in this research study.

What will we do with the research findings?
We will share our findings with the local community of harm reduction providers, Indigenous people who use drugs, academia, and decision-makers through the guideline, reports, possibly manuscripts, and community events and conferences. We will also make our findings available on the Elevate, OAHAS, and CAAN websites.

How will we protect your privacy and confidentiality?
The information you provided in the survey is confidential and private and will be protected in several ways. Your name is not collected and written on either the informed consent form or the survey. We will have your name on a separate form connected to an ID number. This number will be on the consent form and survey. After we are done collecting data we will destroy this form. Some information such as gender, age, city and ethnoracial group may be used to identify you if combined. If your demographic details are unique or obvious, the team will make an effort to exclude it and any information in the survey that might be identifying will not appear in any publication, report, or presentation. Each research team member is responsible for the safekeeping of information and for maintaining privacy and confidentiality. The electronic information will be stored in password protected computer files. Hard copies of digital and written information including consent forms will be kept in a locked cabinet at the WHiSE 2.0 site in your city behind a locked door. The research information will be kept for five years after the study is completed then destroyed using a shredder and professional disposal service. The research team will protect your confidentiality to the extent required by law, there may be limited instances such as when information about harm to a minor is disclosed where the research team has a duty to report this to the authorities in the protection of the child.

The research study you are participating in may be reviewed for quality assurance to make sure that the required laws and guidelines are followed. If chosen, (a) representative(s) of the Human Research Ethics Program (HREP) may access study-related data and/or consent materials as part of the review under the supervision of the nominated PI. All information accessed by the HREP will be upheld to the same level of confidentiality that has been stated by the research team.

What if you want to withdraw?
Your decision to be in the study is completely voluntary, and you are free to drop out at any time. If you drop out, the compensation is yours to keep. The survey will be completed on a tablet so the information you provided up to the last question answered will be kept if we submitted it on the tablet. If we have not yet submitted the data we can delete it. The survey is de-identified which means that a number assigned to your name is located on the signature page. If you choose to drop out or can't show up to a scheduled interview please tell the Research Assistant in your city.

Who can you contact about this research study?
If you have any questions about your rights as a research participant please contact the Research Oversight and Compliance Office - Human Research Ethics Program at 416-946-3273 or ethics.review@utoronto.ca. They are not involved with this research study and calling them will not affect your participation in this research study.

If you have any questions or comments about this study, you can contact Anita C. Benoit who is an Investigator on this project and an Assistant Professor at the Department of Health and Society, University of Toronto Scarborough (647) 601-4566 or anita.benoit@utoronto.ca.

Or in Thunder Bay: Holly Gauvin, Executive Director, Elevate NOW hgauvin@elevatenwo.org

In Sault Ste Marie: Meghan Young, Executive Director, Oahas, Meghan@oahas.org

In Sudbury: Meghan Young, Executive Director, Oahas, Meghan@oahas.org

Participant Informed Consent Form

Project Title: Walking for Harm Reduction through Street Engagement (WHiSE 2.0)

Participant
By signing this form, I confirm that:
• This research study has been fully explained to me and all of my questions answered to my satisfaction
• I understand the requirements of participating in this research study
• I have been informed of the risks and benefits, if any, of participating in this research study
• I have been informed of any alternatives to participating in this research study
• I have been informed of the rights of research participants
• I have read each page of this form
• I authorize access to my personal health information and research study data as explained in

this form
• I have agreed to participate in this study.

Research Assistant Confirmation (Person obtaining consent)
By signing this form, I confirm that:
• This study and its purpose has been explained to the participant named above
• All questions asked by the participant have been answered
• I will give a copy of this signed and dated document to the participant (if requested)

____

Date: (Y/M/D)

____

Participant ID (use numbers only, do not include symbols such as dashes "-")

____

Preamble
I'd like to ask you questions about yourself. Some of the questions in this survey are very personal. Please remember that the answers that you give are completely confidential. We are asking everyone who participates in this survey the same questions. As we said in the informed consent form, the information we will be collecting will be de-identified. This means we will only be able to link the answers to the questions below to you through an ID number and not your name or any other information that could be used to identify you. Thank you for your participation.

Demograpics

1. Can you tell me from which Indigenous grouping(s) you belong to? Select all that apply.

- ☐ First Nations
- ☐ Inuk
- ☐ Métis
- ☐ Any combination of the three
- ☐ Other, please specify:

Please specify "any combination of the three"

____

Please specify "other"

____

---

2. How old are you? Age in years:

____

---

3. What is your current gender identity? Select only one answer.

Gender is not the same as being male or female based on your sex organs, but instead is based on social and cultural differences and the roles that best describe who you feel you are.

- ◯ Woman
- ◯ Trans Woman (male to female)
- ◯ Man
- ◯ Trans Man (female to male)
- ◯ Non-binary (neither man nor woman)
- ◯ Genderqueer
- ◯ 2-Spirit
- ◯ Other, please specify:
- ◯ Don't know
- ◯ Prefer not to answer

Please specify "other"

____

4. What was your sex at birth? Select only one answer.<br><br>Sex at birth and gender are two different concepts but when asked together along with sexual orientation we can get a better understanding of the sexual and gender diversity of participants. This will allow us to make more inclusive and safe recommendations, and reach better conclusions. Your responses will be kept private and secure however, if you feel uncomfortable responding to this question, please select "prefer not to answer".	◯ Male<br>◯ Female<br>◯ Intersex<br>◯ Other, please specify:<br>◯ Don't know<br>◯ Prefer not to answer
Please specify "other"	__________________________________
5. Which of the following best describes your current sexual orientation? Select only one answer.	◯ Heterosexual or Straight<br>◯ Gay<br>◯ Lesbian<br>◯ Bisexual<br>◯ Queer<br>◯ Questioning<br>◯ Other, please specify:<br>◯ Don't know<br>◯ Prefer not to answer
Please specify "other"	__________________________________
6. Do you have a partner(s) right now?	◯ Yes<br>◯ No
7. Is your partner(s) a....? Select all that apply.	☐ Casual partner<br>☐ Regular partner<br>☐ Other, please specify:<br>☐ Don't know<br>☐ Prefer not to answer
Please specify "other"	__________________________________
8. How many of these partners are you currently having sex with? Select only one answer.	◯ None<br>◯ 1 partner<br>◯ 2 partners<br>◯ 3 partners<br>◯ 4 or 5 partners<br>◯ 6 to 10 partners<br>◯ More than 10 partners<br>◯ Don't know<br>◯ Prefer not to answer

9. What is your current relationship status? Select all that apply.	☐ Single<br>☐ Dating<br>☐ Married<br>☐ Consensual non-monogamous (e.g. polyamory, open relationship etc.)<br>☐ Common-law<br>☐ Widowed<br>☐ Separated<br>☐ Divorced<br>☐ Other, please specify:<br>☐ Don't know<br>☐ Prefer not to answer
Please specify "other"	__________________________________
10. Which Indigenous community or territory do you belong to? List all answers. NOTE: This can also mean First Nations reserve, Métis settlement, etc. Select only one answer.	◯ My community, territory, reserve or settlement is:<br>◯ I don't know<br>◯ Prefer not to answer
Please specify "community or territory" (separate the different communities you list with a comma ",")	__________________________________
11. What is your first language? Select only one answer.<br><br>The Indigenous Languages Act was enacted in 2019 and aims to promote and protect the diverse languages spoken by Indigenous people in Canada. We recognize that this list does not include all of the languages spoken in the area but are the majority of languages spoken as a first language or as a preferred language. Please select what you consider your first language, if it is not listed here please specify.	◯ English<br>◯ Ojibway<br>◯ Oji-Cree<br>◯ French<br>◯ Cree<br>◯ Inuktitut<br>◯ Other, please specify:<br>◯ Don't know<br>◯ Prefer not to answer
Please specify "other"	__________________________________
12. What is your preferred language to use? Select only one answer.<br><br>The Indigenous Languages Act was enacted in 2019 and aims to promote and protect the diverse languages spoken by Indigenous people in Canada. We recognize that this list does not include all of the languages spoken in the area but are the majority of languages spoken as a first language or as a preferred language. Please select what you consider your first language, if it is not listed here please specify.	◯ English<br>◯ Ojibway<br>◯ Oji-Cree<br>◯ French<br>◯ Cree<br>◯ Inuktitut<br>◯ Other, please specify:<br>◯ Don't know<br>◯ Prefer not to answer
Please specify "other"	__________________________________

13. What is your current sleeping situation? Select all that apply.

- ☐ House that I own
- ☐ House that I rent
- ☐ Family member's house
- ☐ Family member's apartment
- ☐ Hotel room rented daily
- ☐ Hotel room rented weekly
- ☐ Hotel room rented monthly
- ☐ Friend's house
- ☐ Friend's apartment
- ☐ Shelter
- ☐ On the street
- ☐ Couch surfing
- ☐ Cluster unit/tent city
- ☐ Single room occupancy unit (rooming house)
- ☐ Apartment that I own
- ☐ Apartment that I rent
- ☐ Other, please specify:
- ☐ Don't know
- ☐ Prefer not to answer

Please specify "other" ________________________________

14. Of all the places you indicated sleeping, where have you slept the most in the last three months? Select only one answer.

- ◯ House that I own
- ◯ House that I rent
- ◯ Family member's house
- ◯ Family member's apartment
- ◯ Hotel room rented daily
- ◯ Hotel room rented weekly
- ◯ Hotel room rented monthly
- ◯ Friend's house
- ◯ Friend's apartment
- ◯ Shelter
- ◯ On the street
- ◯ Couch surfing
- ◯ Cluster unit/tent city
- ◯ Single room occupancy unit (rooming house)
- ◯ Apartment that I own
- ◯ Apartment that I rent
- ◯ Other, please specify:
- ◯ Don't know
- ◯ Prefer not to answer

Please specify "other" ________________________________

15. Of all the places other people call home where have you stayed (but not slept) the most in the last three months? Select only one answer.

- ◯ House that I own
- ◯ House that I rent
- ◯ Family member's house
- ◯ Family member's apartment
- ◯ Hotel room rented daily
- ◯ Hotel room rented weekly
- ◯ Hotel room rented monthly
- ◯ Friend's house
- ◯ Friend's apartment
- ◯ Shelter
- ◯ On the street
- ◯ Couch surfing
- ◯ Cluster unit/tent city
- ◯ Single room occupancy unit (rooming house)
- ◯ Apartment that I own
- ◯ Apartment that I rent
- ◯ Other, please specify:
- ◯ Don't know
- ◯ Prefer not to answer

Please specify "other"

____

16. Which city do you currently live in?

- ◯ Thunder Bay
- ◯ Sault Ste. Marie
- ◯ Sudbury

17. On what side of Thunder Bay do you spend most of your time? Select only one answer.

- ◯ Westfort
- ◯ East End
- ◯ Downtown Port Arthur
- ◯ Rural
- ◯ Fort William
- ◯ Intercity
- ◯ Current River
- ◯ Jumbo Gardens
- ◯ Northwood
- ◯ Bay & Algoma
- ◯ County Park
- ◯ Other, please specify:
- ◯ Don't know
- ◯ Prefer not to answer

Please specify "other"

____

18. On what side of Sault Ste. Marie do you spend most of your time? Select only one answer.

◯ Bayview
◯ Broadview Gardens
◯ Brookfield
◯ Buckley
◯ Carpin Beach
◯ Cedar Heights
◯ East Korah
◯ Eastside
◯ Grandview Gardens
◯ Korah
◯ Manitou Park
◯ McQueen
◯ Meadow Park
◯ Nokomis Beach
◯ Odena
◯ The P Patch
◯ Pointe aux Pins
◯ Pointe des Chênes
◯ Pointe Louise
◯ Steelton
◯ Downtown
◯ James Town
◯ West End
◯ East End
◯ Hill Top
◯ Rankin
◯ Fort Creek
◯ Other, please specify
◯ Don't know
◯ Prefer not to answer

Please specify "other"

____

19. On what side of Sudbury do you spend most of your time? Select only one answer.

◯ Downtown
◯ Flour Mill
◯ Bell Park
◯ New Sudbury
◯ South End
◯ West End
◯ Minnow Lake
◯ Donovan/Northern Heights
◯ Gatchell
◯ Copper Cliff
◯ Other, please specify:
◯ Don't know
◯ Prefer not to answer

Please specify "other"

____

20. People earn money in different ways to support themselves. How do you support yourself? Select all that apply.

- ☐ Working full-time
- ☐ Working part-time
- ☐ Supported by family
- ☐ Employment insurance
- ☐ Supported by partner
- ☐ Under the table work
- ☐ Pension/old age security
- ☐ Sex work
- ☐ Panhandling
- ☐ Middling drug transactions
- ☐ Shoplifting
- ☐ Ontario works
- ☐ Ontario Disability Support Program
- ☐ Private healthcare disability Insurance
- ☐ Workers' compensation
- ☐ Selling drugs
- ☐ Honorariums
- ☐ Loansharking
- ☐ Selling items
- ☐ Accepting email money transfers
- ☐ Boosting*
- ☐ Other, please specify:
- ☐ Don't know
- ☐ Prefer not to answer

*Boosting for example is selling knockoff products

Please specify "other"

____

---

21. Indigenous people have been impacted by colonization in many different ways. If you were impacted how were you impacted? Please select all that apply.

- ☐ I have not been impacted by colonization in any way
- ☐ Intergenerational school survivor (i.e., a parent or grandparent went to residential schools)
- ☐ Residential school survivor
- ☐ You were a parent whose child was taken during the sixties scoop
- ☐ You were a child taken during the sixties scoop
- ☐ Involved in the Child Welfare System as a Parent
- ☐ Involved in the Child Welfare System as a Child
- ☐ I was discriminated against when trying to get healthcare
- ☐ Someone I know went missing or was murdered
- ☐ I was discriminated against in my work place
- ☐ I was discriminated against in the justice system
- ☐ I was discriminated against in the education system
- ☐ Other, please specify:
- ☐ Don't know
- ☐ Prefer not to answer

Please specify "other"

____

Culture and Connection

22. Do you practice First Nations, Inuit or Métis ceremonies for healing, celebration or other purposes?

- ◯ Yes
- ◯ No

22.1 If yes, select all answers that apply.

- ☐ Smudging
- ☐ Attending Cultural Groups
- ☐ Drumming
- ☐ Hunting
- ☐ Participating in ceremonies
- ☐ Fishing
- ☐ Singing
- ☐ Sweats
- ☐ Making traditional crafts
- ☐ Attending or participating in pow wows
- ☐ Connecting with Elders and Knowledge Keepers for teachings
- ☐ Learning or speaking my language
- ☐ Making Regalia
- ☐ Other, please specify:
- ☐ Don't know
- ☐ Prefer not to answer

Please specify "other"

____

22.2 If no, why? Select all answers that apply.

- ☐ No access
- ☐ Clash with beliefs
- ☐ Not interested
- ☐ Too many competing things in my life
- ☐ Can't afford it
- ☐ Can't find the right cultural service providers
- ☐ Don't know where to go
- ☐ Lack of trust
- ☐ I don't have knowledge of ceremonies
- ☐ I don't have a connection to ceremonies
- ☐ Stigma
- ☐ Not available
- ☐ Other, please specify:
- ☐ Don't know
- ☐ Prefer not to answer

Please specify "other"

____

23. Do you see reflections of First Nations, Métis or Inuit identity in your community?

- ◯ Yes
- ◯ No

23.1 If yes, where? Please select all answers that apply.

- ☐ Agencies
- ☐ People
- ☐ Advertising
- ☐ Restaurants
- ☐ Media (television, radio, social media)
- ☐ Art
- ☐ Other, please specify:
- ☐ Don't know
- ☐ Prefer not to answer

Please specify "other"

____

24. Where do you find your strength? Select all answers that apply.

- ☐ Connection to culture
- ☐ Religion
- ☐ Creator (interviewer should ask if it's tied to culture)
- ☐ Community or neighbourhood
- ☐ Connection to the land
- ☐ Family
- ☐ God (interviewer should ask if it's tied to religion)
- ☐ Spirituality
- ☐ Other, please specify:
- ☐ Don't know
- ☐ Prefer not to answer

Please specify "other" __________________________________

Drug use history

I'm going to ask you about some of the drugs you use, how you use them and how often you use them. For each drug that you have used, I will ask you if you use daily, more than once per week, once per week, 1-3 times a month, less than once per month or never.

Injection Drugs

25. Have you injected drugs in the last 3 months?

- ◯ Yes
- ◯ No

25.1 If yes, have you injected any of the drugs below in the last 3 months? Read list out and for each drug ask about the frequency of use. Check all responses that apply.

	Less than once a month	1-3 times a month	Once per week	More than once per week	Daily	Never	Not applicable	Prefer not to answer
Heroin	◯	◯	◯	◯	◯	◯	◯	◯
Crystal Meth	◯	◯	◯	◯	◯	◯	◯	◯
Cocaine	◯	◯	◯	◯	◯	◯	◯	◯
Crack Cocaine	◯	◯	◯	◯	◯	◯	◯	◯
Speedball (stimulant mixed with Opioids)	◯	◯	◯	◯	◯	◯	◯	◯
Morphine	◯	◯	◯	◯	◯	◯	◯	◯
Hydromorphone	◯	◯	◯	◯	◯	◯	◯	◯
Generic Oxycodone	◯	◯	◯	◯	◯	◯	◯	◯
Fentanyl	◯	◯	◯	◯	◯	◯	◯	◯
Wellbutrin	◯	◯	◯	◯	◯	◯	◯	◯
Ritalin or Biphentin	◯	◯	◯	◯	◯	◯	◯	◯
Tranquilizers or Benzos	◯	◯	◯	◯	◯	◯	◯	◯

Amphetamines (speed, uppers, dexies, bennies)	◯	◯	◯	◯	◯	◯	◯	◯
Steroids	◯	◯	◯	◯	◯	◯	◯	◯
Gabapentin	◯	◯	◯	◯	◯	◯	◯	◯
Other, specify:	◯	◯	◯	◯	◯	◯	◯	◯

Please specify "other" __________________________________

26. Have you ever injected drugs and...

26.1 consumed alcohol at the same time? Select only one answer.
- ◯ Every time
- ◯ Some of the time
- ◯ Rarely
- ◯ Never
- ◯ Prefer not to answer

26.2 consumed any other products or homemade concoctions (e.g., babash, hairspray, brew, hand sanitizer) at the same time? Select only one answer.
- ◯ Every time
- ◯ Some of the time
- ◯ Rarely
- ◯ Never
- ◯ Prefer not to answer

26.3 Have you ever injected alone?
- ◯ Yes
- ◯ No

26.4 If yes, why did you inject alone? Select all that apply.
- ☐ I didn't want to share
- ☐ I didn't have enough to share
- ☐ I didn't want people to know (I wanted to be discrete)
- ☐ I wanted to see if I could do it on my own
- ☐ I learned how to
- ☐ I was afraid of the stigma
- ☐ I didn't have a spare needle
- ☐ Other, please specify:
- ☐ Don't know
- ☐ Prefer not to answer

Please specify "other" __________________________________

26.5 Do you know about the buddy system? NOTE: The buddy system is when you use with someone else and take turns spotting for each other
- ◯ Yes
- ◯ No

26.6 Do you use the buddy system?
- ◯ Yes
- ◯ No

27. Which part of your body do you prefer to inject? Select only one answer.

◯ Hand
◯ Arm
◯ Neck
◯ Genitals
◯ Leg/Thigh
◯ Between toes
◯ Foot/Feet
◯ Other, please specify:
◯ Don't Know
◯ Prefer not to answer

Please specify "other"

____

28. Have you ever injected with a stranger?

◯ Yes
◯ No
◯ Don't know
◯ Prefer not to answer

29. Have you ever had someone else help you inject?

◯ Yes
◯ No
◯ Don't know
◯ Prefer not to answer

29.1 If yes, why did you want someone to help you inject? Select all that apply.

☐ I was afraid I would waste the drugs
☐ I don't know how to mix the drugs
☐ I don't know how to inject
☐ I was afraid of getting track marks
☐ I was afraid of being alone and high
☐ I was afraid of overdosing
☐ I was afraid of making air bubbles
☐ Other, please specify:
☐ Don't know
☐ Prefer not to answer

Please specify "other"

____

30. Have you ever helped someone inject?

◯ Yes
◯ No
◯ Don't know
◯ Prefer not to answer

30.1 If yes, why did you help someone inject? Select all that apply.

☐ They were afraid to waste the drugs
☐ They didn't know how to mix the drugs
☐ They didn't know how to inject
☐ They were afraid of getting track marks
☐ They were afraid of being alone and high
☐ They were afraid of overdosing
☐ They were afraid of making air bubbles
☐ I wanted to manage the amount I injected them with so there would be some left for me
☐ It was their first time trying drugs
☐ Other, please specify:
☐ Don't know
☐ Prefer not to answer

Please specify "other"

____

31. In which location(s) do you usually inject drugs? Select all that apply.	☐ Home<br>☐ Friend's place<br>☐ Trap house<br>☐ Street/Back lane/laneway<br>☐ Shooting gallery<br>☐ Public bathroom<br>☐ Park<br>☐ Bar/Club<br>☐ Car<br>☐ Train<br>☐ Cluster unit/tent city<br>☐ Other, please specify:<br>☐ Don't know<br>☐ Prefer not to answer
Please specify "other"	__________________________
32. How often do you use the locations you selected above? Select only one answer.	○ Every day<br>○ A few times a week<br>○ Once a week<br>○ Once a month
33. How often do you change the location you inject drugs? Select only one answer.	○ I change locations every time I inject<br>○ I change locations once a day<br>○ I change locations a couple of times a day<br>○ I change locations once a week<br>○ I change locations a couple of times a week<br>○ I change locations once a month<br>○ I change locations a couple of times a month<br>○ I never change locations when I inject<br>○ Other, please specify:<br>○ Don't know<br>○ Prefer not to answer
Please specify "other"	__________________________
33.1 Why did you change the location where you inject drugs? Select all that apply.	☐ I didn't feel safe anymore<br>☐ It became difficult to get to<br>☐ Afraid of getting caught by police<br>☐ The place was raided by police<br>☐ Got kicked out of the space<br>☐ I didn't want to share<br>☐ I only had enough for myself<br>☐ I learned how to inject myself<br>☐ Other, please specify:<br>☐ Don't know<br>☐ Prefer not to answer
Please specify "other"	__________________________

Smoking Drugs

34. Have you smoked drugs other than marijuana in the last 3 months?
- ◯ Yes
- ◯ No

34.1 If yes, have you smoked any of the drugs below in the last 3 months? Read list out and for each drug ask about the frequency of use. Check all responses that apply.

	Less than once a month	1-3 times a month	Once per week	More than once per week	Daily	Never	Not applicable	Prefer not to answer
Heroin	◯	◯	◯	◯	◯	◯	◯	◯
Crystal Meth	◯	◯	◯	◯	◯	◯	◯	◯
Cocaine	◯	◯	◯	◯	◯	◯	◯	◯
Crack Cocaine	◯	◯	◯	◯	◯	◯	◯	◯
Speedball (stimulant mixed with Opioids)	◯	◯	◯	◯	◯	◯	◯	◯
Percocet	◯	◯	◯	◯	◯	◯	◯	◯
Tranquilizers or Benzos	◯	◯	◯	◯	◯	◯	◯	◯
Other, specify:	◯	◯	◯	◯	◯	◯	◯	◯

Please specify "other" ______________________________

35 Have you ever smoked drugs and....

35.1 consumed alcohol at the same time? Select only one answer.
- ◯ Every time
- ◯ Some of the time
- ◯ Rarely
- ◯ Never
- ◯ Prefer not to answer

35.2 consumed any other products or homemade concoctions (e.g., babash, hairspray, brew, hand sanitizer) at the same time? Select only one answer.
- ◯ Every time
- ◯ Some of the time
- ◯ Rarely
- ◯ Never
- ◯ Prefer not to answer

35.3 Have you ever smoked alone?
- ◯ Yes
- ◯ No

35.4 If, yes, why did you smoke alone? Select all that apply.
- ☐ I didn't want to share
- ☐ I didn't have enough to share
- ☐ I didn't want people to know (I wanted to be discrete)
- ☐ I wanted to see if I could do it on my own
- ☐ I learned how to smoke drugs myself
- ☐ I was afraid of the stigma
- ☐ Other, please specify:
- ☐ Don't know
- ☐ Prefer not to answer

Please specify "other" ______________________________

36. In which location(s) do you usually smoke drugs? Select all that apply.

- ☐ Home
- ☐ Friend's place
- ☐ Trap house
- ☐ Street
- ☐ Public bathroom
- ☐ Park
- ☐ Bar/Club
- ☐ Car
- ☐ Train
- ☐ Cluster unit/tent city
- ☐ Other, please specify:
- ☐ Don't know
- ☐ Prefer not to answer

Please specify "other" __________________________________

37. How often do you use the locations you selected above? Select only one answer.

- ◯ Every day
- ◯ A few times a week
- ◯ Once a week
- ◯ Once a month

38. How often do you change the location you smoke drugs? Select only one answer.

- ◯ I change locations every time I smoke
- ◯ I change locations once a day
- ◯ I change locations a couple of times a day
- ◯ I change locations once a week
- ◯ I change locations a couple of times a week
- ◯ I change locations once a month
- ◯ I change locations a couple of times a month
- ◯ I never change locations when I smoke
- ◯ Other, please specify
- ◯ Don't know
- ◯ Prefer not to answer

Please specify "other" __________________________________

38.1 Why did you change the location where you smoke drugs? Select all that apply.

- ☐ I didn't feel safe anymore
- ☐ It became difficult to get to
- ☐ Afraid of getting caught by police
- ☐ The place was raided by the police
- ☐ Got kicked out of the space
- ☐ I didn't want to share
- ☐ I only had enough for myself
- ☐ I learned how to do it myself
- ☐ Other, please specify:
- ☐ Don't know
- ☐ Prefer not to answer

Please specify "other" __________________________________

Oral Drugs

39. Have you taken drugs (orally, swallowing) in the last 3 months?
◯ Yes
◯ No

39.1 If yes, have you taken any of the below drugs in the last 3 months (Read list out. For each drug they have taken ask about the frequency of use. Check responses that apply.)

	Less than once a month	1-3 times a month	Once per week	More than once per week	Daily	Never	Not applicable	Prefer not to answer
Methadone prescribed to you	◯	◯	◯	◯	◯	◯	◯	◯
Methadone not prescribed to you	◯	◯	◯	◯	◯	◯	◯	◯
Morphine	◯	◯	◯	◯	◯	◯	◯	◯
Hydromorphone	◯	◯	◯	◯	◯	◯	◯	◯
Percocet	◯	◯	◯	◯	◯	◯	◯	◯
Generic Oxycodone	◯	◯	◯	◯	◯	◯	◯	◯
Oxy Neo	◯	◯	◯	◯	◯	◯	◯	◯
Fentanyl	◯	◯	◯	◯	◯	◯	◯	◯
Wellbutrin	◯	◯	◯	◯	◯	◯	◯	◯
Ritalin or Biphentin	◯	◯	◯	◯	◯	◯	◯	◯
Tranquilizers or Benzos	◯	◯	◯	◯	◯	◯	◯	◯
Amphetamines (speed, uppers, dexies, bennies)	◯	◯	◯	◯	◯	◯	◯	◯
Valium	◯	◯	◯	◯	◯	◯	◯	◯
Gabapentin	◯	◯	◯	◯	◯	◯	◯	◯
Suboxone prescribed to you	◯	◯	◯	◯	◯	◯	◯	◯
Suboxone not prescribed to you	◯	◯	◯	◯	◯	◯	◯	◯
Other (Specify)	◯	◯	◯	◯	◯	◯	◯	◯

Please specify "other"
____

40 Have you ever orally taken drugs and....?

40.1 consumed alcohol at the same time? Select only one answer.
◯ Every time
◯ Some of the time
◯ Rarely
◯ Never
◯ Prefer not to answer

40.2 consumed any other products or homemade concoctions (e.g., babash, hairspray, brew, hand sanitizer) at the same time? Select only one answer.
◯ Every time
◯ Some of the time
◯ Rarely
◯ Never
◯ Prefer not to answer

40.3 been alone?
◯ Yes
◯ No

40.4 If, yes, why did you take drugs orally alone? Select all that apply.	☐ I didn't want to share<br>☐ I didn't have enough to share<br>☐ I didn't want people to know (I wanted to be discrete)<br>☐ I wanted to see if I could do it on my own<br>☐ I learned how to<br>☐ I was afraid of the stigma<br>☐ Other, please specify:<br>☐ Don't know<br>☐ Prefer not to answer
Please specify "other"	__________________________________
41. In which location(s) do you usually take drugs orally? Select all that apply.	☐ Home<br>☐ Friend's place<br>☐ Trap house<br>☐ Street<br>☐ Public bathroom<br>☐ Park<br>☐ Bar/Club<br>☐ Car<br>☐ Train<br>☐ Cluster unit/tent city<br>☐ Other, please specify:<br>☐ Don't know<br>☐ Prefer not to answer
Please specify "other"	__________________________________
42. How often do you use the locations you selected above? Select only one answer.	○ Every day<br>○ A few times a week<br>○ Once a week<br>○ Once a month
43. How often do you change the location you take drugs orally? Select only one answer.	○ I change locations every time I take drugs orally<br>○ I change locations once a day<br>○ I change locations a couple of times a day<br>○ I change locations once a week<br>○ I change locations a couple of times a week<br>○ I change locations once a month<br>○ I change locations a couple of times a month<br>○ I never change locations when I take drugs orally<br>○ Other, please specify:<br>○ Don't know<br>○ Prefer not to answer
Please specify "other"	__________________________________

43.1 Why did you change the location where you take drugs orally? Select all that apply.

- ☐ I didn't feel safe anymore
- ☐ It became difficult to get to
- ☐ Afraid of getting caught by police
- ☐ The place was raided by police
- ☐ Got kicked out of the space
- ☐ I didn't want to share
- ☐ I only had enough for myself
- ☐ I learned how to take drugs orally myself
- ☐ Other, please specify:
- ☐ Don't know
- ☐ Prefer not to answer

Please specify "other" ______________________________

Sniffing/Snorting/Inhaling Drugs

44. Have you taken drugs by sniffing (snorting, inhaling) in the last 3 months?

- ◯ Yes
- ◯ No

44.1 If yes, have you taken any of the below drugs in the last 3 months (Read list out. For each drug they have taken ask about the frequency of use. Check responses that apply.)

	Less than once a month	1-3 times a month	Once per week	More than once per week	Daily	Never	Not applicable	Prefer not to answer
Solvents (glue, gas, paint thinner etc.)	◯	◯	◯	◯	◯	◯	◯	◯
Whippets/laughing gas (nitrous oxide)	◯	◯	◯	◯	◯	◯	◯	◯
Poppers (nitrites e.g. Rush, Bolt, and Jungle Juice,	◯	◯	◯	◯	◯	◯	◯	◯
Snappers	◯	◯	◯	◯	◯	◯	◯	◯
Room Odourizers/Aromas	◯	◯	◯	◯	◯	◯	◯	◯
Aerosols (hair spray, spray paint, etc.)	◯	◯	◯	◯	◯	◯	◯	◯
Heroin	◯	◯	◯	◯	◯	◯	◯	◯
Cocaine	◯	◯	◯	◯	◯	◯	◯	◯
Bath Salts	◯	◯	◯	◯	◯	◯	◯	◯
Ketamine	◯	◯	◯	◯	◯	◯	◯	◯
Morphine	◯	◯	◯	◯	◯	◯	◯	◯
Hydromorphone	◯	◯	◯	◯	◯	◯	◯	◯
Percocet	◯	◯	◯	◯	◯	◯	◯	◯
Generic Oxycodone	◯	◯	◯	◯	◯	◯	◯	◯
Oxy Neo	◯	◯	◯	◯	◯	◯	◯	◯
Fentanyl	◯	◯	◯	◯	◯	◯	◯	◯

Other (Specify)	○	○	○	○	○	○	○	○

Please specify "other" __________________________________

45. Have you ever sniffed (snorted, inhaled) drugs and....?

45.1 consumed alcohol at the same time? Select only one answer.
- ○ Every time
- ○ Some of the time
- ○ Rarely
- ○ Never
- ○ Prefer not to answer

45.2 consumed any other products or homemade concoctions (e.g., babash, hairspray, brew, hand sanitizer) at the same time? Select only one answer.
- ○ Every time
- ○ Some of the time
- ○ Never
- ○ Rarely
- ○ Prefer not to answer

45.3 Have you ever sniffed (snorted, inhaled) drugs alone?
- ○ Yes
- ○ No

45.4 If, yes, why did you sniff (snort, inhale) drugs alone? Select all that apply.
- ☐ I didn't want to share
- ☐ I didn't have enough to share
- ☐ I didn't want people to know (I wanted to be discrete)
- ☐ I wanted to see if I could do it on my own
- ☐ I learned how to
- ☐ I was afraid of the stigma
- ☐ Other, please specify:
- ☐ Don't know
- ☐ Prefer not to answer

Please specify "other" __________________________________

46. In which location(s) do you usually sniff (snort, inhale) drugs? Select all that apply.
- ☐ Home
- ☐ Friend's place
- ☐ Trap house
- ☐ Street
- ☐ Public bathroom
- ☐ Park
- ☐ Bar/Club
- ☐ Car
- ☐ Train
- ☐ Cluster unit/tent city
- ☐ Other, please specify:
- ☐ Don't know
- ☐ Prefer not to answer

Please specify "other" __________________________________

47. How often do you use the locations you selected above? Select only one answer.
- ○ Every day
- ○ A few times a week
- ○ Once a week
- ○ Once a month

48. How often do you change the location you sniff (snort, inhale) drugs? Select only one answer.

- ◯ I change locations every time I sniff (snort, inhale)
- ◯ I change locations once a day
- ◯ I change locations a couple of times a day
- ◯ I change locations once a week
- ◯ I change locations a couple of times a week
- ◯ I change locations once a month
- ◯ I change locations a couple of times a month
- ◯ I never change locations when I sniff (snort, inhale)
- ◯ Other, please specify:
- ◯ Don't know
- ◯ Prefer not to answer

Please specify "other" ______________________________

48.1 Why did you change the location where you sniff (snort, inhale) drugs? Select all that apply.

- ☐ I didn't feel safe anymore
- ☐ It became difficult to get to
- ☐ Afraid of getting caught by police
- ☐ The place was raided by police
- ☐ Got kicked out of the space
- ☐ I didn't want to share
- ☐ I only had enough for myself
- ☐ I learned how to sniff (snort, inhale) drugs myself
- ☐ Other, please specify:
- ☐ Don't know
- ☐ Prefer not to answer

Please specify "other" ______________________________

Booty Bumping/Hooping

The following set of questions ask about more ways that people use drugs. Some of these questions may be sensitive. Your responses are anonymous.

49. Have you taken drugs by booty bumping/hooping (inserting drugs into the back hole/anus) in the last 3 months?

- ◯ Yes
- ◯ No

49.1 If yes, have you taken any of the below drugs in the last 3 months (Read list out. For each drug they have taken ask about the frequency of use. Check responses that apply.)

	Less than once a month	1-3 times a month	Once per week	More than once per week	Daily	Never	Not applicable	Prefer not to answer
Cocaine	◯	◯	◯	◯	◯	◯	◯	◯
Crystal Meth	◯	◯	◯	◯	◯	◯	◯	◯
Ketamine	◯	◯	◯	◯	◯	◯	◯	◯
Morphine	◯	◯	◯	◯	◯	◯	◯	◯
Hydromorphone	◯	◯	◯	◯	◯	◯	◯	◯

Percocet	◯	◯	◯	◯	◯	◯	◯	◯
Generic Oxycodone	◯	◯	◯	◯	◯	◯	◯	◯
Oxy Neo	◯	◯	◯	◯	◯	◯	◯	◯
Fentanyl	◯	◯	◯	◯	◯	◯	◯	◯
Methadone prescribed to you	◯	◯	◯	◯	◯	◯	◯	◯
Methadone not prescribed to you	◯	◯	◯	◯	◯	◯	◯	◯
Wellbutrin	◯	◯	◯	◯	◯	◯	◯	◯
Ritalin or Biphentin	◯	◯	◯	◯	◯	◯	◯	◯
Tranquilizers or Benzos	◯	◯	◯	◯	◯	◯	◯	◯
Amphetamines (speed, uppers, dexies, bennies)	◯	◯	◯	◯	◯	◯	◯	◯
Valium	◯	◯	◯	◯	◯	◯	◯	◯
Gabapentin	◯	◯	◯	◯	◯	◯	◯	◯
Suboxone prescribed to you	◯	◯	◯	◯	◯	◯	◯	◯
Suboxone not prescribed to you	◯	◯	◯	◯	◯	◯	◯	◯
Other (specify)	◯	◯	◯	◯	◯	◯	◯	◯

Please specify "other" ________________________________

50. Have you ever booty bumped/hooped drugs and.....

50.1 consumed alcohol at the same time? Select only one answer.
- ◯ Every time
- ◯ Some of the time
- ◯ Rarely
- ◯ Never
- ◯ Prefer not to answer

50.2 consumed any other products or homemade concoctions (e.g., babash, hairspray brew, hand sanitizer) at the same time? Select only one answer.
- ◯ Every time
- ◯ Some of the time
- ◯ Rarely
- ◯ Prefer not to answer

50.3 Have you ever booty bumped/hooped drugs alone?
- ◯ Yes
- ◯ No

50. 4 If, yes, why did you booty bumped/hooped drugs alone? Select all that apply.
- ☐ I didn't want to share
- ☐ I didn't have enough to share
- ☐ I didn't want people to know (I wanted to be discrete)
- ☐ I wanted to see if I could do it on my own
- ☐ I learned how to
- ☐ I was afraid of the stigma
- ☐ Other, please specify:
- ☐ Don't know
- ☐ Prefer not to answer

Please specify "other" ________________________________

50.5 Have you ever had anal (back hole) sex within 90 minutes of booty bumping/hooping?
- ◯ Yes
- ◯ No

51. In which location(s) do you usually booty bump/hoop drugs? Select all that apply.

- ☐ Home
- ☐ Friend's place
- ☐ Trap house
- ☐ Street
- ☐ Public bathroom
- ☐ Park
- ☐ Bar/Club
- ☐ Car
- ☐ Train
- ☐ Cluster unit/tent city
- ☐ Other, please specify:
- ☐ Don't know
- ☐ Prefer not to answer

Please specify "other"

____

52. How often do you use the locations you selected above? Select only one answer.

- ◯ Once a day
- ◯ A few times a week
- ◯ Once a week
- ◯ Once a month

53. How often do you change the location you booty bump/hoop drugs? Select only one answer.

- ◯ I change locations every time I booty bump/hoop
- ◯ I change locations once a day
- ◯ I change locations a couple of times a day
- ◯ I change locations once a week
- ◯ I change locations a couple of times a week
- ◯ I change locations once a month
- ◯ I change locations a couple of times a month
- ◯ I never change locations when I booty bump/hoop
- ◯ Other, please specify:
- ◯ Don't know
- ◯ Prefer not to answer

Please specify "other"

____

53.1 Why did you change the location where you booty bump/hoop drugs? Select all that apply.

- ☐ I didn't feel safe anymore
- ☐ It became difficult to get to
- ☐ Afraid of getting caught by police
- ☐ The place was raided by police
- ☐ Got kicked out of the space
- ☐ I didn't want to share
- ☐ I only had enough for myself
- ☐ I learned how to booty bump/hoop drugs myself
- ☐ Other, please specify:
- ☐ Don't know
- ☐ Prefer not to answer

Please specify "other"

____

Plugging Drugs

54. Have you taken drugs by plugging (inserting drugs into the front hole/vagina) in the last 3 months?

◯ Yes
◯ No

54.1 If yes, have you taken any of the below drugs in the last 3 months (Read list out. For each drug they have taken ask about the frequency of use. Check responses that apply.)

	Less than once a month	1-3 times a month	Once per week	More than once per week	Daily	Never	Not Applicable	Prefer not to answer
Cocaine	◯	◯	◯	◯	◯	◯	◯	◯
Crystal Meth	◯	◯	◯	◯	◯	◯	◯	◯
Ketamine	◯	◯	◯	◯	◯	◯	◯	◯
Morphine	◯	◯	◯	◯	◯	◯	◯	◯
Hydromorphone	◯	◯	◯	◯	◯	◯	◯	◯
Percocet	◯	◯	◯	◯	◯	◯	◯	◯
Generic Oxycodone	◯	◯	◯	◯	◯	◯	◯	◯
Oxy Neo	◯	◯	◯	◯	◯	◯	◯	◯
Fentanyl	◯	◯	◯	◯	◯	◯	◯	◯
Methadone prescribed to you	◯	◯	◯	◯	◯	◯	◯	◯
Methadone not prescribed to you	◯	◯	◯	◯	◯	◯	◯	◯
Wellbutrin	◯	◯	◯	◯	◯	◯	◯	◯
Ritalin or Biphentin	◯	◯	◯	◯	◯	◯	◯	◯
Tranquilizers or Benzos	◯	◯	◯	◯	◯	◯	◯	◯
Amphetamines (speed, uppers, dexies, bennies)	◯	◯	◯	◯	◯	◯	◯	◯
Valium	◯	◯	◯	◯	◯	◯	◯	◯
Gabapentin	◯	◯	◯	◯	◯	◯	◯	◯
Suboxone prescribed to you	◯	◯	◯	◯	◯	◯	◯	◯
Suboxone not prescribed to you	◯	◯	◯	◯	◯	◯	◯	◯
Other (Specify)	◯	◯	◯	◯	◯	◯	◯	◯

Please specify "other"

____

55. Have you ever plugged drugs and...

55.1 consumed alcohol at the same time? Select only one answer.

◯ Every time
◯ Some of the time
◯ Rarely
◯ Never
◯ Prefer not to answer

55.2 consumed any other products or homemade concoctions (e.g., babash, hairspray , brew, hand sanitizer) at the same time? Select only one answer.

◯ Every time
◯ Some of the time
◯ Rarely
◯ Never
◯ Prefer not to answer

55.3 Have you ever plugged drugs alone?	◯ Yes<br>◯ No
55.4 If, yes, why did you plug drugs alone? Select all that apply.	☐ I didn't want to share<br>☐ I didn't have enough to share<br>☐ I didn't want people to know (I wanted to be discrete)<br>☐ I wanted to see if I could do it on my own<br>☐ I learned how to<br>☐ I was afraid of the stigma<br>☐ Other, please specify:<br>☐ Don't know<br>☐ Prefer not to answer
Please specify "other"	__________________________________
55.5 Have you ever had vaginal/front hole sex within 90 minutes of plugging?	◯ Yes<br>◯ No
56. In which location(s) do you usually plug drugs? Select all that apply.	☐ Home<br>☐ Friend's place<br>☐ Trap house<br>☐ Street<br>☐ Public bathroom<br>☐ Park<br>☐ Bar/Club<br>☐ Car<br>☐ Train<br>☐ Cluster unit/tent city<br>☐ Other, please specify:<br>☐ Don't know<br>☐ Prefer not to answer
Please specify "other"	__________________________________
57. How often do you use the locations you selected above? Select only one answer.	◯ Every day<br>◯ A few times a week<br>◯ Once a week<br>◯ Once a month
58. How often do you change the location you plug drugs? Select only one answer.	◯ I change locations every time I plug drugs<br>◯ I change locations once a day<br>◯ I change locations a couple of times a day<br>◯ I change locations once a week<br>◯ I change locations a couple of times a week<br>◯ I change locations once a month<br>◯ I change locations a couple of times a month<br>◯ I never change locations when I plug drugs<br>◯ Other, please specify:<br>◯ Don't know<br>◯ Prefer not to answer
Please specify "other"	__________________________________

58.1 Why did you change the location where you plug drugs? Select all that apply.

- ☐ I didn't feel safe anymore
- ☐ It became difficult to get to
- ☐ Afraid of getting caught by police
- ☐ The place was raided by police
- ☐ Got kicked out of the space
- ☐ I didn't want to share
- ☐ I only had enough for myself
- ☐ I learned how to plug drugs myself
- ☐ Other, please specify:
- ☐ Don't know
- ☐ Prefer not to answer

Please specify "other"

____

The following questions are about drug use in general.

59. How do you usually get your drugs? Select all that apply.

- ☐ I buy my drugs from a seller
- ☐ I buy my drugs from my main seller
- ☐ I buy drugs from another person who uses drugs
- ☐ I use a middle person to buy drugs
- ☐ I sell drugs and get paid in drugs
- ☐ I exchange work for drugs
- ☐ I exchange sex for drugs
- ☐ I exchange food or shelter for drugs
- ☐ I share drugs with a partner or a friend
- ☐ I have a trusted source who buys it for me
- ☐ I have a sugar daddy/sugar mommy
- ☐ Other, please specify:
- ☐ Don't know
- ☐ Prefer not to answer

Please specify "other"

____

60. Have you ever used more than one drug at a time?

- ◯ Yes
- ◯ No

60.1 If yes, how do you mix the different drugs that you use? Select all that apply.

- ☐ I crush the drugs and mix them for injecting
- ☐ I crush the drugs and mix them for smoking
- ☐ I crush the drugs and mix them for swallowing orally
- ☐ I crush the drugs and mix them for sniffing/snorting
- ☐ I crush the drugs and mix them for booty bumping/hooping (inserting in back hole/anus)
- ☐ I crush the drugs and mix them for plugging (inserting in front hole/vagina)
- ☐ I sniff/snort different lines of crushed drugs (crisscrossing)
- ☐ Other, please specify:
- ☐ Don't know
- ☐ Prefer not to answer

____

61. What is your preferred drug to use?

____

61.1 Is this the drug that is available to you the most often?	◯ Yes<br>◯ No
61.2 Is this drug the easiest to get?	◯ Yes<br>◯ No
62. Have you ever overdosed?	◯ Yes<br>◯ No<br>◯ Not sure<br>◯ Don't know<br>◯ Prefer not to answer
63. When was your most recent overdose?	◯ Within the last week<br>◯ Within the last month<br>◯ Within the last 3 months<br>◯ Within the last 4-5 months<br>◯ Within the last 6-12 months<br>◯ 1 Year ago or more<br>◯ Don't know<br>◯ Prefer not to answer
64. How many times have you overdosed in your lifetime?	◯ Once<br>◯ Twice<br>◯ Three times<br>◯ Four times<br>◯ Five times<br>◯ More than five times<br>◯ Don't know<br>◯ Prefer not to answer
65. Have you ever witnessed someone else overdose?	◯ Yes<br>◯ No<br>◯ Not Sure<br>◯ Don't Know<br>◯ Prefer not to answer

Harm Reduction

66. Do you know what harm reduction is? [Ask respondent to explain it and write answer down. If no explain harm reduction to respondent)	◯ Yes<br>◯ No
Respondent's explanation of harm reduction:	__________________________________

If no, interviewer's explanation of harm reduction: Harm reduction is a set of practical strategies and ideas aimed at reducing negative consequences associated with drug use. Harm Reduction is also a movement for social justice built on a belief in, and respect for, the rights of people who use drugs.

67. Can you provide examples of harm reduction?	__________________________________
68. Do you know where to go for harm reduction services and supplies?	◯ Yes<br>◯ No<br>◯ Don't Know<br>◯ Prefer not to answer

Which city do you currently live in?

- ◯ Thunder Bay
- ◯ Sault Ste. Marie
- ◯ Sudbury

68.1 If yes, where are some of the locations a person can access harm reduction in your city? List the other locations that the respondents may not have identified.

- ☐ Elevate NWO
- ☐ PACE
- ☐ Shelter House
- ☐ Health Unit
- ☐ Joseph Esquega Health Centre
- ☐ Superior Points
- ☐ OAHAS Thunder Bay
- ☐ Nor'west Community Health Centre
- ☐ Other, please specify:
- ☐ Don't know
- ☐ Prefer not to answer

Please specify "other" ______________________________

If "No" or 'Don't Know', list the locations where a person can access harm reduction. See list provided for potential answers. RA should share pamphlets and other resources or a list of locations

68.1 If yes, where are some of the locations a person can access harm reduction in your city? List the other locations that the respondents may not have identified.

- ☐ Maamwesying North Shore Community Health Services
- ☐ Sault Area Hospital (SAH) CMHA Peer Program
- ☐ HARP (HIV/AIDS Resource Program) Group Health Centre
- ☐ OAHAS Sault Ste. Marie
- ☐ Algoma Public Health
- ☐ SOYA - Save Our Young Adults
- ☐ A NEW LINK
- ☐ Recovery North
- ☐ Hep Care Team
- ☐ Other, please specify:
- ☐ Don't know
- ☐ Prefer not to answer

Please specify "other" ______________________________

If "No" or 'Don't Know', list the locations where a person can access harm reduction. See list provided for potential answers. RA should share pamphlets and other resources or a list of locations

68.1 If yes, where are some of the locations a person can access harm reduction in your city? List the other locations that the respondents may not have identified.

- ☐ The Point Elm Place
- ☐ The Point Espanola Mall
- ☐ The Point Manitoulin Island
- ☐ Sudbury Action Centre for Youth
- ☐ Réseau ACCESS Network
- ☐ OAHAS Sudbury
- ☐ Public Health Sudbury & Districts
- ☐ Other, please specify:
- ☐ Don't know
- ☐ Prefer not to answer

Please specify "other" ______________________________

If "No" or 'Don't Know', list the locations where a person can access harm reduction. See list provided for potential answers. RA should share pamphlets and other resources or a list of locations

69. Have you ever used the Ellie Van? (Ellie was a street- based outreach van from Elevate NWO that offered harm reduction, testing and support in the community. The van ran from (2017- 2020)

◯ Yes
◯ No

69.1 Would you use the Ellie van if its services resume?

◯ Yes
◯ No

70. What types of in-building services or resources would you like to see delivered to meet your harm reduction needs?

____

70.1 What types of mobile or outreach services would you like to see delivered to meet your harm reduction needs

____

71. Are there things that get in the way of you accessing harm reduction services and supplies?

◯ Yes
◯ No

71.1 If yes, what are some of the things that get in the way of you accessing harm reduction services and supplies? Select all answers that apply.

☐ Transportation
☐ Needing childcare
☐ Privacy
☐ Confidentiality
☐ Not knowing location of sites
☐ Dislike staff
☐ Being barred
☐ Safety
☐ Other, please specify:
☐ Don't know
☐ Prefer not to answer

Please specify "other"

____

72. Are there things that get in the way of you practicing harm reduction?

◯ Yes
◯ No

72.1 If yes, what are some of the things that get in the way of you practicing harm reduction? Select all answers that apply.

☐ Transportation
☐ Needing childcare
☐ Privacy
☐ Confidentiality
☐ Not knowing location of sites
☐ Dislike staff
☐ Cultural or other belief systems
☐ Safety
☐ Being barred
☐ Conflict
☐ Other, please specify:
☐ Don't know
☐ Prefer not to answer

Please specify "other"

____

73. Is there anything about 'teachings' that you received from your family, religious groups, or cultural people that affect how you feel about using harm reduction practices?

◯ Yes
◯ No

73.1 If yes, what are some of the things that affect your ability to practice harm reduction and your teachings at the same time? Select all that apply.	☐ I have to wait four days after having used drugs before going to a sweat<br>☐ I can't participate in ceremony when I'm under the influence of drugs<br>☐ I can't go to church when I'm under the influence of drugs<br>☐ Other, please specify:<br>☐ Don't know<br>☐ Prefer not to answer
Please specify "other"	__________________________

Supervised Injection Site (SIS)

Preamble

Problematic substance use has devastating impacts across Canada, on individuals, families and communities. It is a complex issue. There is no simple solution. Supervised Injection Sites or SIS are part of a harm reduction approach to the Canadian drugs and substances strategy. This is because Canadian and international evidence shows that these sites help save lives and improve health (Health Canada, 2022). Research also shows that SIS are cost effective and do not increase drug use and crime in the surrounding area (Ng, Sutherland & Kolber, 2017). SIS are an entry point to treatment and social services for people who are ready to stop or reduce their use of substances. They do not provide drugs or substances to people that come to the sites. People will use SIS for several reasons. For example:

- a safe, clean place to consume illegal substances
- less risk of violence or confrontation with police
- drug checking to detect adulterants using methods such as fentanyl test strips
- An adulterant is any substance that's added to drugs on purpose and it can make the drug more effective, less effective, easier to use, or it can help increase the amount of drugs and can make drugs unsafe
- emergency medical care in case of overdose, cardiac arrest or allergic reaction (anaphylaxis)
- basic health services, such as wound care
- testing for infectious diseases like HIV, Hepatitis C and Sexually Transmitted and Blood-borne Infections (STBBIs)
- access to sterile drug use equipment and a place to safely dispose of it after use
- health professionals and support staff, including for overdose intervention

74. SIS Services are a part of some local strategies, in which city do you normally access services?	◯ Thunder Bay<br>◯ Sault Ste Marie<br>◯ Sudbury
75. A SIS was opened in the winter of 2018 at the Nor'West Community Health Centre in Thunder Bay were you aware of this?	◯ Yes<br>◯ No
76. Have you used the SIS at the Nor'West Community Health Centre?	◯ Yes<br>◯ No
76.1 If no, describe why:	__________________________
77. Are there things about the SIS that would help you to not use where you normally do and go there instead?	◯ Yes<br>◯ No
77.1 If yes, what are the things you think would support you or another person hold off on using drugs until you can get to the SIS?	__________________________

77.2 If no, what are some of the things that would stop you or another person from holding off on using drugs until you get to a SIS?	__________________________________
78. If a SIS were to exist in a different location in Thunder Bay, would you go there?	◯ Yes<br>◯ No
78.1 If no, what are some of the reasons why you would not go there?	☐ I don't want to be seen at a SIS<br>☐ I go to a friend's place<br>☐ I go to a shooting gallery<br>☐ I use on the streets<br>☐ I use at my home<br>☐ I can't wait<br>☐ Afraid I'll get sick<br>☐ I'm worried about encountering the cops<br>☐ Other, please specify:<br>☐ Don't know<br>☐ Prefer not to answer
Please specify "other"	___________________________
79. If a SIS was opened in Sault Ste. Marie would you use their services?	◯ Yes<br>◯ No
80. Are there things about SIS in general that would help you not use where you normally do and go to an SIS instead if it existed?	◯ Yes<br>◯ No
80.1 If yes, what are some of the things you think would support you or another person hold off on using drugs if you could get to an existing SIS?	__________________________________
80.2 If no, what are some of the things that would stop you or another person from holding off on using drugs even if an SIS existed?	__________________________________
81. If a SIS was opened in Sudbury would you use their services?	◯ Yes<br>◯ No
82. Are there things about SIS in general that would help you to not use where you normally do and go there instead if it existed?	◯ Yes<br>◯ No
82.1 If yes, what are some of the things you think would support you or another person hold off on using drugs if you could get to an existing SIS?	__________________________________
82.2 If no, what are some of the things that would stop you or another person from holding off on using drugs even if an SIS existed?	__________________________________

Supervised Consumption Site (SCS)

Preamble
The next few questions are about supervised consumption sites or SCS. These are similar to SIS which I explained earlier, but are not limited to only one type of consumption like injection drug use in an SIS. Some sites allow injection, oral, vaporized, or intranasal forms of consumption. SCS also have a network of support services on site for individuals such as addiction treatment programs as well as counselling and support for permanent housing.

83. A SCS was opened in the summer of 2022 at the Réseau ACCESS Network's Safe Consumption Site in Sudbury located at Energy Court were you aware of this?
- ◯ Yes
- ◯ No

84. Have you used the SCS at the Réseau ACCESS Network's Safe Consumption Site in Sudbury located at Energy Court?
- ◯ Yes
- ◯ No

84.1 If no, describe why:

____

85. Are there things about the SCS that would help you to not use where you normally do and go there instead?
- ◯ Yes
- ◯ No

85.1 If yes, what among all the things you listed would support you or another person hold off on using drugs until you can get to the SCS?

____

85.2 If no, what are some of the things that would stop you or another person from holding off on using drugs until you get to a SCS?

____

86. If a SCS were to exist in a different location in Sudbury, would you go there?
- ◯ Yes
- ◯ No

86.1 If no, what are some of the reasons why you would not go there? Select all that apply.
- ☐ I don't want to be seen at a SCS
- ☐ I go to a friend's place
- ☐ I go to a shooting gallery
- ☐ I use on the streets
- ☐ I use at my home
- ☐ I can't wait
- ☐ I'm worried about encountering the cops
- ☐ Afraid I'll get sick
- ☐ Other, please specify
- ☐ Don't know
- ☐ Prefer not to answer

Please specify "other"

____

87. If a SCS were to exist in Thunder Bay, would you go there?
- ◯ Yes
- ◯ No

87.1 If yes, describe why:

____

87.2 If no, select the reasons why:	☐ I don't want to be seen at a SCS<br>☐ I go to a friend's place<br>☐ I go to a shooting gallery<br>☐ I use on the streets<br>☐ I use at my home<br>☐ I can't wait<br>☐ I'm worried about encountering the cops<br>☐ Afraid I'll get sick<br>☐ Other, please specify:<br>☐ Don't know<br>☐ Prefer not to answer
Please specify "other"	__________________________
88. If a SCS were to exist in Sault Ste Marie, would you go there?	◯ Yes<br>◯ No
88.1 If yes, describe why:	________________________________
88.2 If no, select the reasons why:	☐ I don't want to be seen at a SCS<br>☐ I go to a friend's place<br>☐ I go to a shooting gallery<br>☐ I use on the streets<br>☐ I use at my home<br>☐ I can't wait<br>☐ I'm worried about encountering the cops<br>☐ Afraid I'll get sick<br>☐ Other, please specify:<br>☐ Don't know<br>☐ Prefer not to answer
Please specify "other"	__________________________
89. As an Indigenous person what ...	
89.1 would you like to see or have in a SIS/SCS to make you feel safer when accessing it?	________________________________
89.2 would help you feel like you belong and are supported in a SIS/SCS?	________________________________
89.3 services need to be in place to support you to come to an SIS/SCS (select all answers that apply)?	☐ Indigenous counselling services<br>☐ Non-judgemental staff<br>☐ Family support<br>☐ Sharing/support circles<br>☐ Holistic care<br>☐ Traditional healers<br>☐ Short wait times<br>☐ Peers working there<br>☐ Other, please specify:<br>☐ Don't know<br>☐ Prefer not to answer

Please specify "other" ______________________

89.4 services need to be in place to support you to come back again to a SIS/SCS, to become a returning client? Select all that apply.

- ☐ Food
- ☐ Access to Elders
- ☐ Bus tickets
- ☐ Drug testing strips
- ☐ Clothing/furniture donations
- ☐ ID clinic
- ☐ Advocacy workers
- ☐ Short wait times
- ☐ Other, please specify:
- ☐ Don't know
- ☐ Prefer not to answer

Please specify "other" ______________________

89.5 would a caring staff look like or do at SIS/SCS? Select all that apply.

- ☐ Respect privacy
- ☐ Staff that don't make you wait a long time to talk or meet
- ☐ Fight for client rights
- ☐ Know the people that they serve
- ☐ Have lived experience
- ☐ Other, please specify:
- ☐ Don't know
- ☐ Prefer not to answer

Please specify "other" ______________________

90. What would a SIS/SCS have to do to be inclusive of your needs and values? ______________________

Health

Preamble
The First Nations and Inuit Health Branch (FNIHB), the Assembly of First Nations (AFN), and Indigenous mental health leaders from various First Nations community organizations worked together to create the First Nations Mental Wellness Continuum Framework which focuses on strengths and resilience and understands how purpose, hope, belonging and meaning through culture can impact the lives of people across their lifespan. The framework describes key services that all Indigenous communities should have access to.

91. Which of the following mental wellness services have you been able to access?

	Yes	No	Don't Know	Not applicable	Prefer not answer
Health Promotion, Prevention, Community Development and Education (Oahas, Elevate NWO, ONWA)	○	○	○	○	○
Early Identification and Intervention	○	○	○	○	○

Crisis Response	◯	◯	◯	◯	◯
Coordination of Care and Care Planning	◯	◯	◯	◯	◯
Detox	◯	◯	◯	◯	◯
Trauma-informed Treatment	◯	◯	◯	◯	◯
Support and Aftercare	◯	◯	◯	◯	◯
Elders and Traditional Knowledge Keepers	◯	◯	◯	◯	◯
Traditional Healers	◯	◯	◯	◯	◯

92. Was the mental wellness service you received delivered in a culturally safe manner?

	Yes	No	Don't Know	Not Applicable	Prefer not to answer
Health Promotion, Prevention, Community Development and Education (Oahas, Elevate NWO, ONWA)	◯	◯	◯	◯	◯
Early Identification and Intervention	◯	◯	◯	◯	◯
Crisis Response	◯	◯	◯	◯	◯
Coordination of Care and Care Planning	◯	◯	◯	◯	◯
Detox	◯	◯	◯	◯	◯
Trauma-informed Treatment	◯	◯	◯	◯	◯
Support and Aftercare	◯	◯	◯	◯	◯
Elders and Traditional Knowledge Keepers	◯	◯	◯	◯	◯
Traditional Healers	◯	◯	◯	◯	◯

93. Have you ever had substance use related injuries or illness (physical or mental)?

- ◯ Yes
- ◯ No
- ◯ Not Sure
- ◯ Don't Know
- ◯ Prefer not to answer

94. If yes, what type of physical injuries/illness did you experience? Select all that apply.

- ☐ Breathing difficulties
- ☐ Loss of sense of smell
- ☐ Damaged septum (the bony part of your nose that divides into the right and left side)
- ☐ Vomiting
- ☐ Constipation
- ☐ Shakiness
- ☐ Dizziness
- ☐ Increased heart rate
- ☐ Heart failure
- ☐ Coma
- ☐ Seizures
- ☐ Runny nose
- ☐ Frequent cold/allergy symptoms
- ☐ Chronic sinus/nasal problems
- ☐ Nosebleeds
- ☐ Sores on mouth
- ☐ Sores on nose
- ☐ Problems swallowing
- ☐ Bacterial infections of the blood vessels and heart valves
- ☐ Liver disease
- ☐ Kidney disease
- ☐ Pneumonia
- ☐ Tuberculosis
- ☐ Hepatitis B
- ☐ Hepatitis C
- ☐ HIV /AIDS
- ☐ Traumatic brain injury
- ☐ Skin abscess (closed sore or lesion filled with pus)
- ☐ Skin/hand burns
- ☐ Skin Ulcers (open sores or lesions with no pus)
- ☐ Open wounds
- ☐ None of the above
- ☐ Other, please specify:
- ☐ Don't know
- ☐ Prefer not to answer

Please specify "other"

____

95. What type of mental illness/symptoms did you experience? Select all that apply.

- ☐ Depression
- ☐ Anxiety
- ☐ Bipolar Disorder
- ☐ Psychosis
- ☐ Delirium
- ☐ Delusional Disorder
- ☐ Dementia
- ☐ Amnesia
- ☐ Schizophrenia
- ☐ Attention Deficit-Hyperactivity Disorder (ADHD)
- ☐ Paranoia
- ☐ Hallucinations
- ☐ Distrust
- ☐ None of the above
- ☐ Other, please specify:
- ☐ Don't know
- ☐ Prefer not to answer

Please specify "other"

____

96. Have you ever used services for substance use related physical or mental injuries/illness (e.g., care for injection spots, nose bleeds, mental health)	◯ Yes<br>◯ No<br>◯ Don't Know<br>◯ Prefer not to answer
97. If yes, which of the following services have you used? Select all that apply.	☐ Traditional Healer<br>☐ Emergency room<br>☐ Urgent Care<br>☐ Walk-in clinic<br>☐ Street health service<br>☐ Mental health centre or hospital<br>☐ Supervised consumption site<br>☐ AIDS Service Organization (ASO)<br>☐ Community Health Centre<br>☐ Sexual Health Clinic<br>☐ Pharmacy<br>☐ Other, please specify:<br>☐ Don't know<br>☐ Prefer not to answer
Please specify "other"	______________________________

Social Provisions

Preamble
In answering the next set of questions I am going to ask you, I want you to think about your current relationship with friends, family members, coworkers, community members, and so on.

98. Please tell me to what extent you agree that each statement describes your current relationships with other people. I'll ask you to give your opinion using a scale that asks if you strongly agree, agree, disagree or strongly disagree. So, for example, if you feel a statement is very true of your current relationships, you would tell me "strongly agree". If you feel a statement clearly does not describe your relationships, you would respond "strongly disagree". Do you have any questions?

	Strongly Disagree	Disagree	Agree	Strongly Agree
1. There are people I can depend on to help me if I really need it.	◯	◯	◯	◯
2. I feel that I do not have close personal relationships with other people.	◯	◯	◯	◯
3. There is no one I can turn to for guidance in times of stress.	◯	◯	◯	◯
4. There are people who depend on me for help.	◯	◯	◯	◯
5. There are people who enjoy the same social activities I do.	◯	◯	◯	◯
6. Other people do not view me as competent.	◯	◯	◯	◯

7. I feel personally responsible for the well-being of another person.	○	○	○	○
8. I feel part of a group of people who share my attitudes and beliefs.	○	○	○	○
9. I do not think other people respect my skills and abilities.	○	○	○	○
10. If something went wrong, no one would come to my assistance.	○	○	○	○
11. I have close relationships that provide me with a sense of emotional security and well-being.	○	○	○	○
12. There is someone I could talk to about important decisions in my life.	○	○	○	○
13. I have relationships where my competence and skills are recognized.	○	○	○	○
14. There is no one who shares my interests and concerns.	○	○	○	○
15. There is no one who really relies on me for their well-being.	○	○	○	○
16. There is a trustworthy person I could turn to for advice if I were having problems.	○	○	○	○
17. I feel a strong emotional bond with at least one other person.	○	○	○	○
18. There is no one I can depend on for aid if I really need it.	○	○	○	○
19. There is no one I feel comfortable talking about problems with.	○	○	○	○
20. There are people who admire my talents and abilities.	○	○	○	○
21. I lack a feeling of intimacy with another person.	○	○	○	○
22. There is no one who likes to do the things I do.	○	○	○	○
23. There are people I can count on in an emergency.	○	○	○	○

24. No one needs me to care for them. ○ ○ ○ ○

Testing and overdose prevention

99. Have you obtained safer supply material(s) in the past 3 months?

- ○ Yes
- ○ No
- ○ Don't Know
- ○ Prefer not to answer

99.1 If yes, which safer supply materials did you want? Select all that apply.

- ☐ Needle and syringe
- ☐ Cooker
- ☐ Filter
- ☐ Ascorbic acid (Vitamin C)
- ☐ Sterile water distribution
- ☐ Alcohol swab
- ☐ Tourniquet/Arm Band/Towel
- ☐ Tin Foil
- ☐ Safer crack cocaine/crystal meth smoking equipment
- ☐ Sterile straws for sniffing/snorting/inhaling
- ☐ Safer drug use education materials
- ☐ Handling and disposal of used drug use equipment
- ☐ Overdose prevention: education
- ☐ Overdose prevention: naloxone distribution
- ☐ Other, please specify:
- ☐ Don't know
- ☐ Prefer not to answer

Please specify "other" ______________________

99.2 If yes, which safer supply materials were you offered? Select all that apply.

- ☐ Needle and syringe
- ☐ Cooker
- ☐ Filter
- ☐ Ascorbic acid (Vitamin C)
- ☐ Sterile water distribution
- ☐ Alcohol swab
- ☐ Tourniquet/Arm Band/Towel
- ☐ Tin Foil
- ☐ Safer crack cocaine/crystal meth smoking equipment
- ☐ Sterile straws for sniffing/snorting/inhaling
- ☐ Safer drug use education materials
- ☐ Handling and disposal of used drug use equipment
- ☐ Overdose prevention: education
- ☐ Overdose prevention: naloxone distribution
- ☐ Other, please specify:
- ☐ Don't know
- ☐ Prefer not to answer

Please specify "other" ______________________

99.3 If yes, which safer supply materials did you use? Select all that apply.	☐ Needle and syringe<br>☐ Cooker<br>☐ Filter<br>☐ Ascorbic acid (Vitamin C)<br>☐ Sterile water distribution<br>☐ Alcohol swab<br>☐ Tourniquet/Arm Band/Towel<br>☐ Tin Foil<br>☐ Safer crack cocaine/crystal meth smoking equipment<br>☐ Sterile straws for sniffing/snorting/inhaling<br>☐ Safer drug use education materials<br>☐ Handling and disposal of used drug use equipment<br>☐ Overdose prevention: education<br>☐ Overdose prevention: naloxone distribution<br>☐ Other, please specify:<br>☐ Don't know<br>☐ Prefer not to answer
Please specify "other"	______________________
99.4 Did you have to buy any safer supply materials?	◯ Yes<br>◯ No
99.5 If yes, which ones did you have to buy? Select all that apply.	☐ Needle and syringe<br>☐ Cooker<br>☐ Filter<br>☐ Ascorbic acid (Vitamin C)<br>☐ Sterile water distribution<br>☐ Alcohol swab<br>☐ Tourniquet/Arm Band/Towel<br>☐ Tin Foil<br>☐ Safer crack cocaine/crystal meth smoking equipment<br>☐ Sterile straws for sniffing/snorting/inhaling<br>☐ Safer drug use education materials<br>☐ Handling and disposal of used drug use equipment<br>☐ Overdose prevention: education<br>☐ Overdose prevention: naloxone distribution<br>☐ Other, please specify:<br>☐ Don't know<br>☐ Prefer not to answer
Please specify "other"	______________________
100. Have you ever been tested for HIV? (Irrespective of answer let them know that there is always someone onsite if they are interested in getting tested for the first time or again.)	◯ Yes<br>◯ No
100.1 Have you ever been diagnosed with HIV/AIDS?	◯ Yes<br>◯ No
100.2 Have you received treatment for HIV?	◯ Yes<br>◯ No
101. Have you ever been tested for hepatitis C?	◯ Yes<br>◯ No
101.1 Have you ever been diagnosed with hepatitis C?	◯ Yes<br>◯ No

101.2 Have you received treatment for hepatitis C?	◯ Yes<br>◯ No<br>◯ Don't Know<br>◯ Prefer not to answer
102. Have you ever been tested for other sexually transmitted and blood borne infections?	◯ Yes<br>◯ No<br>◯ Don't Know<br>◯ Prefer not to answer
102.1 Have you ever been diagnosed with other sexually transmitted and blood borne infections ?	◯ Yes<br>◯ No<br>◯ Don't Know<br>◯ Prefer not to answer
102.2 Which sexually transmitted and blood borne infections were you diagnosed with?	__________________________________
102.3 Have you received treatment for other sexually transmitted and blood borne infections?	◯ Yes<br>◯ No<br>◯ Don't Know<br>◯ Prefer not to answer
103. Have you ever heard of methadone? [If no, explain what Methadone is (Methadone is a medication primarily used to treat chronic pain and as a treatment for opioid use disorder)]	◯ Yes<br>◯ No
104. Do you use methadone?	◯ Yes<br>◯ No
105. Where do you get your methadone?	◯ Substance use treatment services/clinics (inpatient or outpatient)<br>◯ Pharmacy<br>◯ Social services agency<br>◯ Mental Health Agency/Clinic<br>◯ Community-based health centre/clinic<br>◯ Hospital<br>◯ Doctor's office<br>◯ Mobile dispensing units (e.g. vans),<br>◯ Correctional facilities<br>◯ AIDS Service Organization<br>◯ Prefer not to answer<br>◯ Other, please specify:<br>◯ Don't know<br>◯ Prefer not to answer
Please specify "other"	____________________________
106. Have you ever heard of Naloxone? [If yes, Ask if they need a refresher, if no, explain what Naloxone is (Naloxone hydrochloride (naloxone) is a drug that can temporarily stop the effects of opioid drugs. Naloxone can help restore breathing during an opioid overdose.)]	◯ Yes<br>◯ No
107. Do you have a Naloxone kit? [If yes, ask if they need another kit, if no ask them if they want a kit]	◯ Yes<br>◯ No

107.1 If yes, where did you get your Naloxone kit? Select all that apply.	☐ Ellie Van or other mobile clinic<br>☐ Pharmacy<br>☐ Nurse<br>☐ Friend had it<br>☐ Organization<br>☐ Outreach worker<br>☐ Other, please specify:<br>☐ Don't know<br>☐ Prefer not to answer
Please specify "other"	______________________
108. Were you trained on how to use a Naloxone kit?<br><br>If yes, [Ask if they need a refresher on how to use the kit and let them know that there is someone on site that can provide training.]<br><br>If no, [Ask them it they want to be trained on how to use a kit. Let them know that there is someone on site that can provide training.]	◯ Yes<br>◯ No
109. Have you ever had to use Naloxone?	◯ Yes<br>◯ No
109.1 If yes, how did it come as? Select all that apply.	☐ Injectable<br>☐ Spray
109.2 If yes, did you call the ambulance?<br><br>*Provide a list of where a person can get, and be trained to use naloxone in Thunder Bay, Sault Ste, Marie and Sudbury	◯ Yes<br>◯ No
110. Were you aware of the Good Samaritan Drug Overdose Act?	◯ Yes<br>◯ No

Preamble

I am going to read a simple version of the Good Samaritan Drug Overdose Act from the Canada.gc.ca website. The Good Samaritan Drug Overdose Act provides some legal protection for people who experience or witness an overdose and call 9-1-1 for help. The act can protect you from:

Charges for possession of a controlled substance (i.e. drugs) under section 4(1) of the Controlled Drugs and Substances Act Breach of conditions regarding simple possession of controlled substances (i.e. drugs) in: pre-trial release probation orders conditional sentences parole The Good Samaritan Drug Overdose Act applies to anyone seeking emergency support during an overdose, including the person experiencing an overdose. The act protects the person who seeks help, whether they stay or leave from the overdose scene before help arrives. The act also protects anyone else who is at the scene when help arrives. The act does not provide legal protection against more serious offences, such as:

outstanding warrants production and trafficking of controlled substances all other crimes not outlined within the act

111. Given that we have the Good Samaritan Drug Overdose Act would you be more willing to call 911 if you witnessed an overdose?	◯ Yes<br>◯ No

111.1 If no, describe why:

____

111.2 If drugs were decriminalized would it make you more likely to use the good Samaritan ACT and call 911? Select only one answer.

- ◯ Yes
- ◯ No
- ◯ Don't know
- ◯ Prefer not to answer

111.3 Do you agree with the decriminalization of substances? Select only one answer.

- ◯ Yes
- ◯ No
- ◯ Don't know
- ◯ Prefer not to answer

111.4 In what ways do you think decriminalization might impact your life?

____

Delayed Substance Use

112. When was the last time you used substances?

____

113. Would you be willing to delay before using a substance? Select only one answer.

- ◯ Yes
- ◯ No
- ◯ Don't know
- ◯ Prefer not to answer

113.1 If yes, how long would you be willing to delay before using a substance?

____

113.2 If yes, is there a particular time of day when you are more likely to delay?

____

113.3 If no, what are the reasons you wouldn't delay using a substance?

____

Strength based question

114. What are some of the harm reduction skills that you practice for yourself or for others that you can share with us? Select all that apply.

- ☐ Getting new needles/pipes for other people
- ☐ Getting new needles/pipes for yourself
- ☐ Sharing food with someone that is hungry
- ☐ Carrying condoms
- ☐ Disposing needles in sharps containers
- ☐ Wearing a seatbelt in a vehicle
- ☐ Walking someone home at night
- ☐ Using the same dealer
- ☐ Calling street outreach services (SOS) for somebody
- ☐ Trying a small amount before using the full amount
- ☐ Other, please specify:
- ☐ Don't know
- ☐ Prefer not to answer

Please specify "other"

____

Self-determination

Preamble
The United Nations Charter (Article 1, paragraph 2) describes self-determination as a state having the right to freely choose its political, economic, social, and cultural systems. I'll be asking you some questions about what this means to you.

115. If there were more cultural activities around your neighbourhood would you have more support in general?

- ◯ Yes
- ◯ No
- ◯ Don't Know
- ◯ Prefer not to answer

116. If there were more cultural activities around your neighbourhood would you have more support with using drugs?

- ◯ Yes
- ◯ No
- ◯ Don't Know
- ◯ Prefer not to answer

117. How important is it to you that Indigenous communities manage and control their own....

	Absolutely Essential	Very important	Of average importance	Of little importance	Not important at all
Health programs and services	◯	◯	◯	◯	◯
Research and data	◯	◯	◯	◯	◯
Education systems	◯	◯	◯	◯	◯
Environmental resources (land, fishing rights, food sovereignty, mining rights, environmental safety)	◯	◯	◯	◯	◯
Economic resources (oil and gas, tourism, trade, arts and entertainment)	◯	◯	◯	◯	◯
Housing	◯	◯	◯	◯	◯
Self-governance	◯	◯	◯	◯	◯

118. Is there anything else that you think is important for us to know that we didn't ask about?

- ◯ Yes
- ◯ No

118.1 If yes, please let us know:

____

119. Would you be interested in participating in a focus group about this same topic? An Elder will conduct ceremony and provide support to the participants and research staff in the focus groups. We will explore some of the questions we asked in the survey.

- ◯ Yes
- ◯ No
- ◯ Don't Know
- ◯ Prefer not to answer

Completed interview - Thank participant and remind them of how they can reach you.
