# Supplementary material for: Cohort profile: Walking for harm reduction through street engagement (WHiSE 2.0)
Source: PLoS One. 2026 Jul 24;21(7):e0354477. doi: 10.1371/journal.pone.0354477 (PMC13399352; doi:10.1371/journal.pone.0354477)
Supplement: S2 File — (DOCX) [file pone.0354477.s002.docx]

Inclusivity in global research

PLOS’ policy on inclusivity in global research aims to improve transparency in the reporting of research performed outside of researchers’ own country or community and ensures that PLOS publications reporting global research adhere to high standards for research ethics and authorship. Authors of relevant research articles may be asked to complete the questionnaire below, which outlines ethical, cultural, and scientific considerations specific to inclusivity in global research. This questionnaire may be requested when researchers have travelled to a different country to conduct research, if research uses samples collected in another country, research with Indigenous populations or their lands, or if research is on cultural artefacts. Researchers travelling to another country solely to use laboratory equipment will not normally be required to complete the questionnaire. However, the questionnaire can be requested at the journal’s discretion for any submission – if you have been requested to complete this questionnaire by the PLOS journal you submitted to, please do so.

Please complete the questionnaire below and include this as a Supporting Information file with your manuscript. Note that if your paper is accepted for publication, this checklist will be published with your article in the supporting information files. Please ensure that you reference the checklist in the main body of your manuscript. We suggest adding a subsection ‘Inclusivity in global research’ to your Methods section and adding the following sentence: “Additional information regarding the ethical, cultural, and scientific considerations specific to inclusivity in global research is included in the Supporting Information (SX Checklist)”

The questions have been designed to be applicable to a wide range of study types, and there are subsections for both human subjects research and non-human subjects research. If any of the questions are not relevant to your research please mark them as “N/A” as appropriate.

**Ethical considerations, permits and authorship**

*This section is applicable to all research types.*

Provide details as to who granted permissions and/or consent for the study to take place in the Methods section of your manuscript. This should include the names of **all** ethics boards, governmental organizations, community leaders or other bodies that provided approval for the study. If individuals provided approval refer to these people by their role or title but do not list their name(s).

Reported on page number: Page 12 of 47, line 256-257.

“Research ethics clearance was obtained from the University of Toronto Research Ethics Board (Protocol # 43512). All participants provided verbal and written informed consent.”

Reported on page number: Page 34 of 47, line 632-635. This was reported in accordance with the journal’s instructions in an ethics declaration section.

If there were any deviations from the study protocol after approval was obtained please provide details of these changes in the Methods section of your manuscript.

Reported on page number: n/a.

Did this study involve local collaborators that are residents of the country where the research was conducted or members of the community studied? If you do not have any authors from said communities, please provide an explanation for this below.

The study was conducted in Northern Ontario with local research assistants and local community investigators (organization representatives). They are listed as co-authors or in the acknowledgement section based on their ability to meet the CRediT taxonomy and journal’s policy. The community investigators’ organizations are described in the methods section, page 15 of 47, line 149-160.

Everyone listed as an author should meet PLOS’ criteria for authorship and all individuals who meet these criteria should be included in the author byline, rather than the acknowledgements. For further information please see the journal’s Authorship Policy.

**Human subjects research (e.g. health research, medical research, cross-cultural psychology)**

Did you obtain written informed consent from a representative of the local community or region before the research took place? How did you establish who speaks for the community? Details of written informed consent obtained from study participants should be reported separately in the Methods section of your manuscript.

The local community organization representatives (Executive Directors) did not provide informed consent before the research took place. This study stemmed from their interest and their request to conduct the research study, page 5 of 47. They are principal investigators on the grant and contributed to conceptualizing the study, from study design to research design. Informed consent by study participants is written in the methods section, page 15 of 47, line 257.

How did members of the local community provide input on the aims of the research investigation, its methodology, and its anticipated outcome(s)?

| They are community investigators (principal investigators) on the grant. They determined the study priorities and provided feedback on all aspects of the study from study design (e.g., research question and objectives, prospective study design) to research design (e.g., recruitment, sampling, data collection and analysis, knowledge sharing strategies). The team had several meetings up to submission of the grant. Following success of the grant, the research team meets every 2 months virtually and every month, study updates and priorities are shared over email for discussion which includes local research assistant feedback. Study outputs are approved by the community investigators and a meeting separate from monthly updates was held focused on sharing study outputs to participants and service providers locally. |
| --- |

When engaging with the local community, how did you ensure that the informed consent documents and other materials could be understood by local stakeholders?

| The informed consent form was written and community partners provided feedback on language and length. The local research assistants also indicated whether there was a lack of clarity and changes were made based on these comments. Next the consent form received feedback from the REB as well and the form was modified accordingly. Local research assistants went over the consent form with study participants and answered all their questions. The participants were also offered a hard copy to take and reflect on their interest in participating or if they wanted to share it with someone trusted to discuss.  Study recruitment material was reviewed in a similar manner and was limited to one-page. |
| --- |

Will the findings of the research be made available in an understandable format to stakeholders in the community where the study was conducted (e.g. via a presentation, summary report, copies of publications, etc.)? Please provide details of how this will be achieved.

| Yes, over the course of the study, infographics are shared with the community investigators and the local research assistants to share with participants. They are also posted on social media. We have also requested other local interest-holders to share with us, the research team, if there is specific data they are interested in the team exploring to create a lay language resource.  We are also in the process of writing manuscripts. We have created presentations to share data with our community partners. We continue to create infographics. We have recently held a knowledge sharing meeting to discuss planning in-person events at each of the study sites to share findings with study participants and local staff of the community partners. |
| --- |

**Non-human subjects research using specimens/ animals collected as part of the study, or those housed in archival collections. Examples include archaeology, paleontology, botany and zoology.**

Did the permission you obtained from a local authority to perform the study include an agreement on access to outputs and benefit sharing? This may include procedures to enable fair distribution of the benefits and resources arising from the research performed. Please include any details of Prior Informed Consent and Benefit Sharing Agreements obtained. These may be required by field-specific regulations, for example the Convention on Biological Diversity (CBD) and the associated Nagoya Protocol.

No, we do not have an agreement. We do have a living document that includes information on collaborative manuscript writing and our implementation of the SHARE and OCAP principles (Indigenous data governance principles).

If the material used in your study was imported, please A) provide the year it was imported and B) indicate whether permits were obtained to import/export the materials used, C) provide details of any permits obtained. If this information is not available, please indicate this.

| Not applicable. Our data was not imported.  We are creating a local database of Indigenous use of harm reduction practices and substance use. |
| --- |

If you used archival specimens, please state how the material used in your study was acquired by the institute it is held in and provide details of any permits obtained for the original excavations/ sample collection. If this information is not available, please indicate this.

| Not applicable. |
| --- |

How was the potential cultural significance of the materials collected in your study to local communities considered in your research design? Were Indigenous peoples and/or local researchers and institutions involved with archaeological excavations / collection of specimens? If so, please provide a description of their involvement.

| We are collecting self-reported data using a questionnaire. Our questionnaire stemmed from a prior study that was completed and led by our community investigators. This study is the second iteration that has been expanded to include 3 study sites in Northern Ontario. The questionnaire was discussed in several rounds of sharing circles and focus groups where applicable to update and shape the questionnaire (e.g., length, language, structure/skip patterns, answer options, Indigenous content and appropriateness). |
| --- |

If your manuscript includes photographs of human remains please indicate whether authors obtained permission from descendants or affiliated cultural communities to do so.

Our manuscript does not include any photographs.
